# The effect of alterations of schizophrenia-associated genes on gamma band oscillations

**DOI:** 10.1038/s41537-022-00255-7

**Published:** 2022-04-28

**Authors:** Christoph Metzner, Tuomo Mäki-Marttunen, Gili Karni, Hana McMahon-Cole, Volker Steuber

**Affiliations:** 1 Neural Information Processing Group, Institute of Software Engineering and Theoretical Computer Science, Technische Universität Berlin, Berlin, Germany; 2 Biocomputation Research Group, School of Physics, Engineering and Computer Science, University of Hertfordshire, Hatfield, United Kingdom; 3 Faculty of Medicine and Health Technology, Tampere University, Tampere, Finland; 4 Minerva Schools at KGI, San Francisco, CA USA

**Keywords:** Schizophrenia, Schizophrenia, Biomarkers, Neural circuits

## Abstract

Abnormalities in the synchronized oscillatory activity of neurons in general and, specifically in the gamma band, might play a crucial role in the pathophysiology of schizophrenia. While these changes in oscillatory activity have traditionally been linked to alterations at the synaptic level, we demonstrate here, using computational modeling, that common genetic variants of ion channels can contribute strongly to this effect. Our model of primary auditory cortex highlights multiple schizophrenia-associated genetic variants that reduce gamma power in an auditory steady-state response task. Furthermore, we show that combinations of several of these schizophrenia-associated variants can produce similar effects as the more traditionally considered synaptic changes. Overall, our study provides a mechanistic link between schizophrenia-associated common genetic variants, as identified by genome-wide association studies, and one of the most robust neurophysiological endophenotypes of schizophrenia.

## Introduction

The search for biological causes of psychiatric disorders has up to now met with limited success. While genetics and basic neuroscience have both made tremendous advances over the last decade, mechanistic links between genetic findings and clinical symptoms have so far not been discovered. Many have argued that symptom-based classifications of psychiatric illnesses might not be possible to map to alterations at the microscopic scale^1,2^, and have proposed to use biomarkers or endophenotypes, which in turn might correlate more clearly with genetic variants^2^. For example, the biomarkers and endophenotypes of schizophrenia (SCZ) include reduced mismatch negativity^3^, reduced pre-pulse inhibition^4^ and changes to evoked and induced oscillations in multiple frequency bands in a large variety of tasks (e.g.^5^). Importantly, recent advances in computational modeling allow for the integration of knowledge about genetic contributions to ion channels and excitability and can be used to predict changes to macroscopic electroencephalography (EEG) or magnetoencephalography (MEG) signals^6–8^, (for a review of this emerging subfield of computational psychiatry see ref. ^9^). For example, simulations of a detailed model of tufted layer 5 pyramidal cells have recently been used to predict the effect of SCZ-associated variants of ion channel-encoding genes on neural activity in the delta frequency band^10^.

In general, oscillations in the low and high frequency range enable coordinated interactions between distributed neuronal responses^11–14^ and have been demonstrated to be functionally relevant^15^. For example, gamma oscillations have been linked to perception^16^, attention^17^, memory^18^, consciousness^19^ and synaptic plasticity^20^. In patients with schizophrenia, gamma power and coherence have been consistently found to be decreased during neural entrainment in auditory steady-state response (ASSR) tasks^21–24^ as well as during several sensory (e.g. visual Gestalt^25^) and cognitive (e.g. working memory^26^) tasks. In ASSR neural entrainment tasks, deficits in power and coherence in the gamma band are the most robust and reliable abnormality in patients with schizophrenia^24^. Importantly, schizophrenia is also associated with disturbances in many of the above mentioned functions^27–29^. Mathematical analyses and computer simulations have demonstrated that gamma oscillations arise through the local interplay between excitatory and inhibitory populations, either through tonic excitation of inhibitory cells and subsequent rhythmic inhibition of excitatory cells (interneuron gamma or ING) or through rhythmic excitation of inhibitory cells and subsequent rhythmic inhibition of excitatory cells (pyramidal-interneuron gamma or PING)^30,31^. The anatomical and electrophysiological properties of a particular subtype of inhibitory interneurons, the parvalbumin-positive (PV^+^) interneurons, make them ideally suited for the fast, strong and temporally precise inhibition necessary for the generation of gamma rhythms^32^. Furthermore, optogenetically driving PV^+^ interneurons was found to enhance gamma rhythms^33^. Consequently, cellular level alterations at PV^+^ interneurons in schizophrenia have been linked to well-known ASSR deficits in the gamma band^22,34–37^. However, these studies have focused on changes to the strength and temporal dynamics of synaptic transmission. While synaptic transmission dynamics undoubtedly play a crucial role in the generation of neural oscillations, cell-intrinsic properties such as ionic conductances can also alter the ability of a network to generate and maintain oscillations. This is of particular importance since many of the recently discovered gene variants associated with schizophrenia relate to ionic channels or Ca^2+^ transporters^38,39^.

In this study, we use an established framework to translate the effect of common single-nucleotide polymorphism (SNP) variants associated with schizophrenia into a biophysically detailed model of a layer 5 pyramidal cell^8^. We then use a morphologically reduced version of the cell^40^ together with a model of a PV^+^ interneuron^41^ in a microcircuit model and explore the effect of the genetic variants on gamma entrainment. We specifically focus on entrainment in the gamma band because gamma ASSR deficits have been found to be the most robust ASSR deficit in schizophrenia^24^, because gamma oscillations seem to be generated locally in cortex^42–44^, and because the mechanisms underlying the generation of gamma oscillations have been extensively studied^30^. This allows for a detailed and accurate model of a very robust disturbance.

We demonstrate that while single gene variants typically only have small effects on gamma auditory steady-state entrainment, combinations of them can reduce the entrainment comparable to the synaptic alterations mentioned above and replicate observations in schizophrenia patients. Our findings therefore provide a mechanistic link between the scale of single genes and an important endophenotype of schizophrenia. Furthermore, the proposed model represents an ideal test-bed for the identification of targets for potential pharmacological agents aiming to reverse gamma deficits in schizophrenia.

## Results

**Fig. 1:**
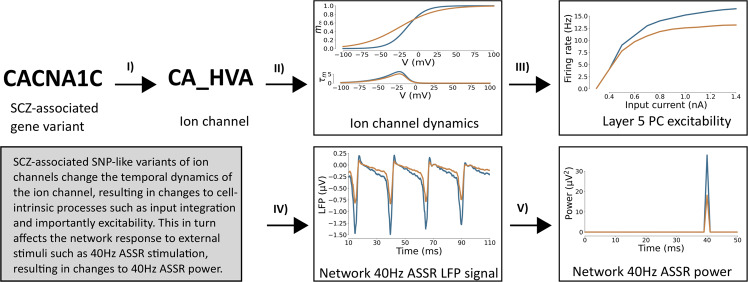
Overview of the effect of genetic variants. Schematic of the cascade of effects from SCZ-associated SNP-like variants changing ion channels (I) to changes in the temporal dynamics of the ion channel (II) to altered excitability of Layer 5 pyramidal cells (III), which in turn changes network-level LFP signals in response to 40Hz ASSR input (IV) effectively changing the gamma band (40Hz) ASSR power (V). Control network depicted in blue, SCZ-associated SNP-like variant in yellow.

We executed simulations of the network model with background synaptic noise and a periodic drive at 40 Hz, mimicking auditory steady-state stimulation experiments (Fig. 7). For each model variant in Supplementary Table 1, we repeated the set of 200 simulations (10 ’trials’ for each of the 20 ’virtual subjects’, see Supplementary Section 1.1.2). For each of these model variants, the parameters of the ion-channels were altered in a subtle way, leading to changes in their activation, and subsequently to modified network dynamics and gamma entrainment (see Fig. 1 for an overview of the approach). For more information see the Methods section and the Supplementary Material.

### Single variants can produce ASSR power deficits in the gamma range

**Fig. 2:**
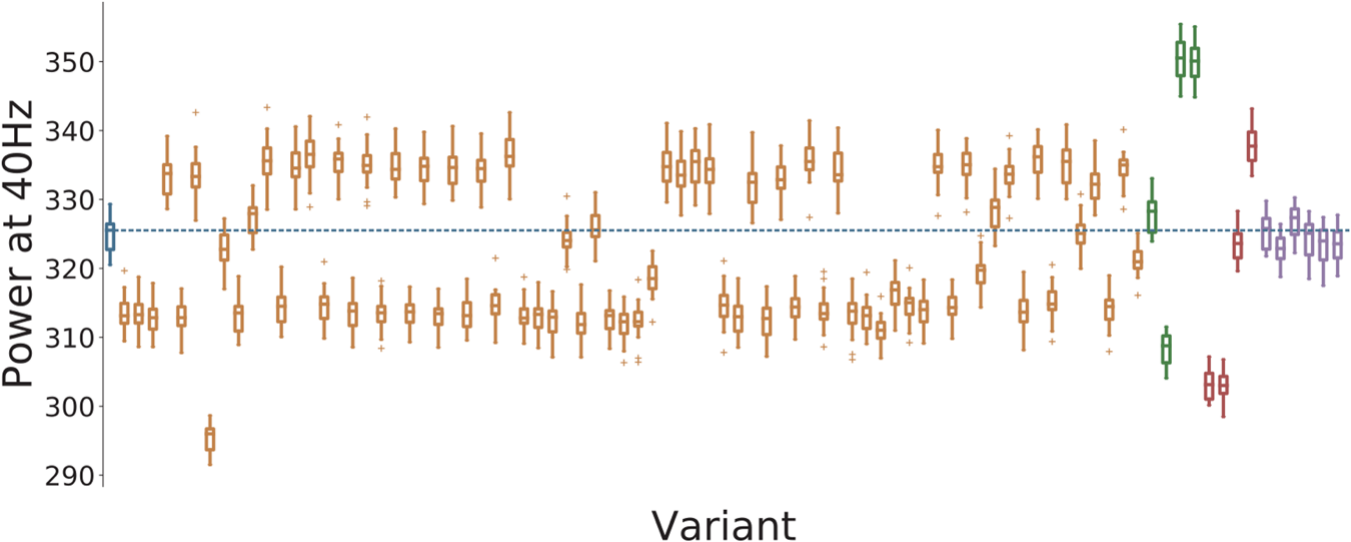
Overview of the 4040 measure for all variants. Control network in blue, blue dashed line represents the mean of the control group, *Ca*^2+^ channel variants affecting Ca_*HVA*_ in orange, *Ca*^2+^ channel variants affecting Ca_*LVA*_ in green, HCN variants in red, and SCN variants in purple. For each color-coded group of variants the ordering matches the ordering in Supplementary Tables 3, 4 and 5, respectively. Solid lines represent the mean, box edges the 25 and 75 percentile, respectively, the whisker extend to 2 standard deviations and + depict outliers. The dashed blue line represents the mean of the control network.

**Fig. 3:**
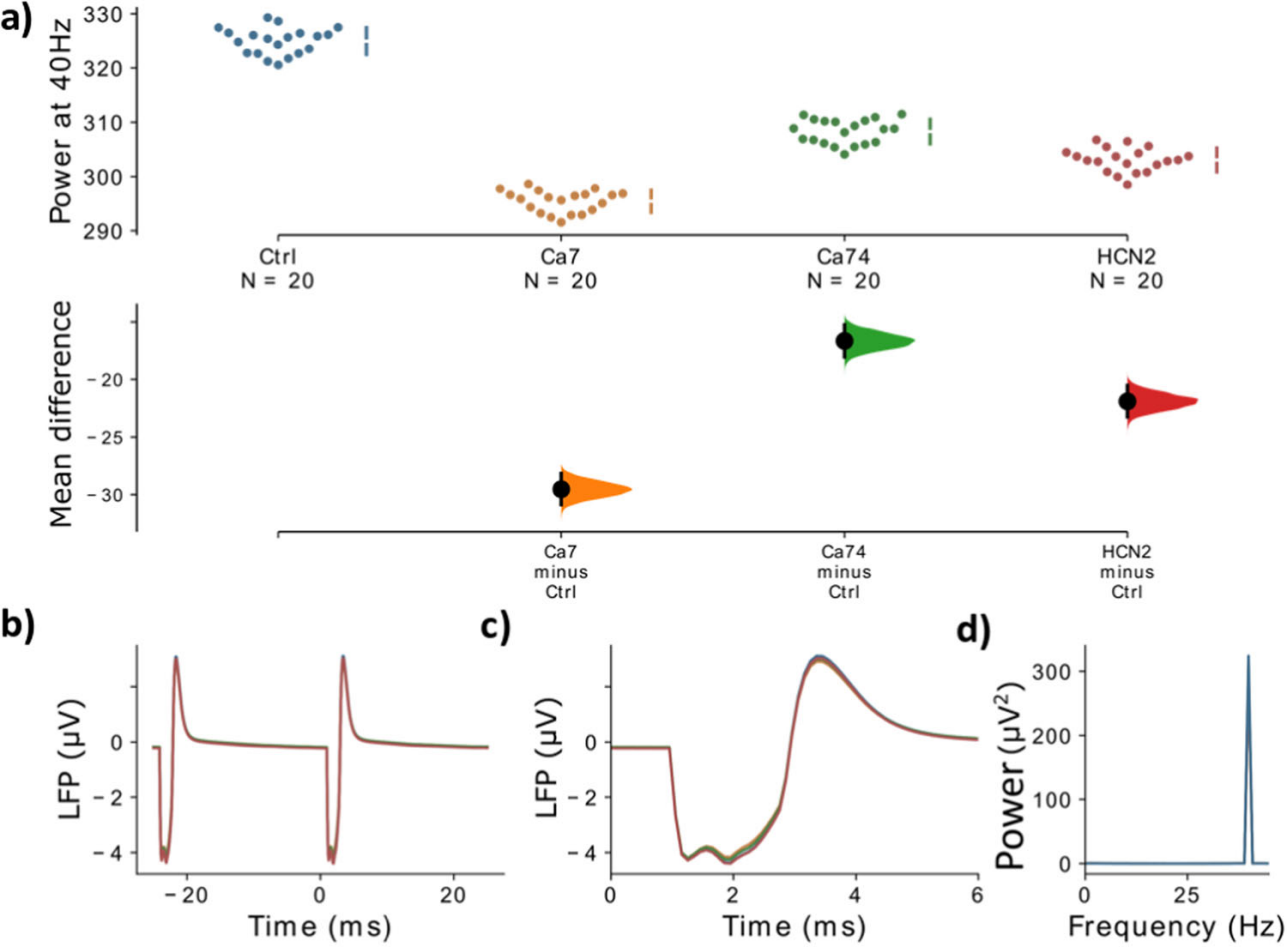
Comparison of different variants against the control. **a** The mean difference, i.e. for three comparisons (the Ca^2+^ channel model variants, Ca7 and Ca74, and the HCN model variant, HCN1-2, with the strongest gamma reduction) against the shared control are shown in the above Cumming estimation plot. The raw data is plotted on the upper axes. On the lower axes, mean differences are plotted as bootstrap sampling distributions. Each mean difference is depicted as a dot. Each 95% confidence interval is indicated by the ends of the vertical error bars. **b** The simulated LFP signal for the control network (blue), Ca7 (yellow), Ca74 (green), and HCN1-2 (red) is shown, averaged over two consecutive gamma cycles. **c** The two signals from **b** are presented with a zoom into the narrow time frame after the stimulus arrives at 0 ms. **d** The power spectral density (PSD) for the LFP signals from **b**. Note that in **b**–**d** the LFP signal is first averaged over all ‘subjects’ and ‘trials’.

First, we analyzed the evoked ASSR power in the gamma range. Most of the model variants altered gamma power in a weak or moderate way and the model variants could both increase or decrease gamma power (Fig. 2). Model variants affecting the *Na*^+^ channels had hardly any effect on entrainment power which reflects the small scaling coefficients imposed by the downscaling scheme (see Supplementary Table 3). Four model variants, one affecting the CACNA1C gene, one affecting the CACNA1I gene and two affecting the HCN1A gene, led to strong decreases of evoked power (Fig. 3). For the model variants affecting *I*_*CaHVA*_, the change in evoked gamma power was positively correlated with the offset of the activation variable *m* (Pearson correlation: *r* = 0.53, *p* < 0.001; Supplementary Fig. 3) but there was no significant correlation with other parameters describing the ion channel dynamics (Supplementary Fig. 3). Furthermore, no model variant strongly increased the 20 Hz component (see Supplementary Fig. 2), i.e. we observed no shift to the first subharmonic of the 40 Hz drive, which would be indicative of a ’beat-skipping’ behavior, where the inhibition suppresses every other drive stimulus as seen in models of altered synaptic dynamics^22^. In our implementation of the click train stimuli we assumed that the click train input arrives at the model neurons simultaneously. This results in clear and narrow peaks in the simulated LFP signal. While this makes the analysis of the observed effects very easy it is not completely realistic. In Supplementary Fig. 6, we present a comparison of the control network with the ’Ca7’ model variant in a ’jittered’ condition, where the input for each neuron is jittered by a few milliseconds, creating a more realistic scenario. We found the same effect of the model variant on gamma power as in the ’non-jittered’ condition demonstrating the robustness of our findings.

### Combinations of variants can produce substantial ASSR power deficits

**Fig. 4:**
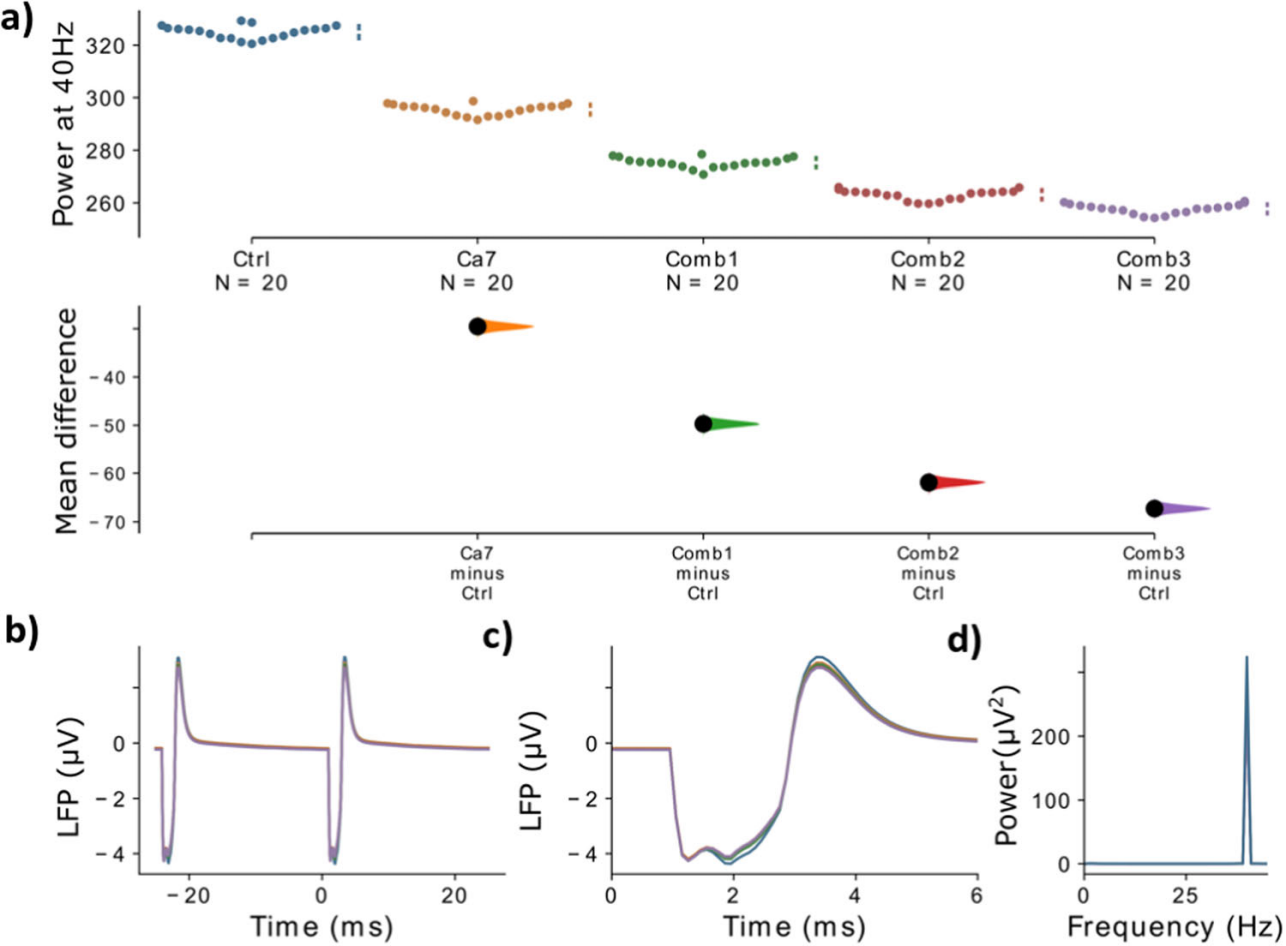
Comparison of different combinations of variants against the control. **a** The mean difference for the comparisons of three variant combinations and the single variant with the strongest effect (Ca7) against the shared control are shown in the above Cumming estimation plot. The raw data is plotted on the upper axes. On the lower axes, mean differences are plotted as bootstrap sampling distributions. Each mean difference is depicted as a dot. Each 95% confidence interval is indicated by the ends of the vertical error bars. **b** The simulated LFP signal for the control network (blue), Ca7 (yellow), combination 1 (green), combination 2 (red) and combination 3 (purple) is shown, averaged over two consecutive gamma cycles. **c** The signals from **b** are presented with a zoom into the narrow time frame after the stimulus arrives at 0 ms. **d** The power spectral density (PSD) for the LFP signals from **b**. Note that in **b**–**d** the LFP signal is first averaged over all ‘subjects’ and ‘trials’.

Next, we tested three different combinations of model variants (details in Supplementary Tables 2 and 3). The combinations were: (1) the combination of the two model variants with the strongest gamma reduction from the single model variant trials (Ca7 affecting gene CACNA1C and HCN1-2 affecting HCN1; this combination will be referred to as *Comb1*); (2) the model variants from (1) but additionally one more model variant with a moderate gamma reduction (Ca74 affecting CACNA1I gene, this combination will be referred to as *Comb2*); (3) the model variants from Comb2 plus an additional model variant with a moderate gamma reduction (HCN1-1 affecting the HCN1 gene; this combination will be referred to as *Comb3*)). Note here that both model variants HCN1-1 and HCN1-2 had similar effects, i.e. they both negatively shifted the offset and increased the slope of parameter *m* of the *I*_*h*_ channel. In the last case, when combining model variants of the same gene, we assume a linear superposition of the effects of the single model variants on the parameters of the ion channels. Note that this is a simplistic assumption and that there could potentially be nonlinear interactions between different variants of the same gene. However, actual experimental data on this relationship are currently not available. We found that combining model variants further increased their effect on evoked gamma power and a combination of only a few variants already had a strong impact on gamma power (Fig. 4 and Supplementary Table 3). Overall, the effects of combining model variants were additive, e.g. combining the two model variants Ca7 and HCN1-2 which individually have a mean difference of −29.5 and −21.9 results in a combination with a mean difference of −49.7 which is roughly the sum of the two individual mean differences (see also Supplementary Table 4).

### The effects of variant combinations are comparable to the effects of synaptic alterations

Changes to the GABAergic system have been proposed to explain the reduction in evoked gamma power^32^ and modeling work has demonstrated that both, a reduction in GABA levels in schizophrenia patients^34,45^ and an increase in decay times at GABAergic synapses^22,34^, can lead to reduced ASSR gamma power. On the one hand, a decrease in inhibition due to reduced GABA levels decreases the precise inhibitory control over pyramidal cell firing necessary for a strong gamma rhythm. On the other hand, an increase in GABAergic decay times, while increasing inhibition, can lead to suppression of pyramidal cell firing every second gamma cycle (a phenomenon called ’beat-skipping’) and thus strongly reduce gamma power. Therefore, we compared the model variant effects to the effects of these two changes to the GABAergic system of the network. Specifically, we implemented a 25% reduction of the maximum conductance of GABAergic synapses (see e.g. ref. ^34^) and an increase of the decay time of inhibitory postsynaptic currents (IPSCs) at GABAergic synapses from 8 ms to 25 ms (e.g. refs. ^22,34^). These two conditions will be referred to as *Gmax* and *IPSC*. Comparing the effects of the model variants and their combinations to these two changes allowed us to judge the relative size of their effect.

**Fig. 5:**
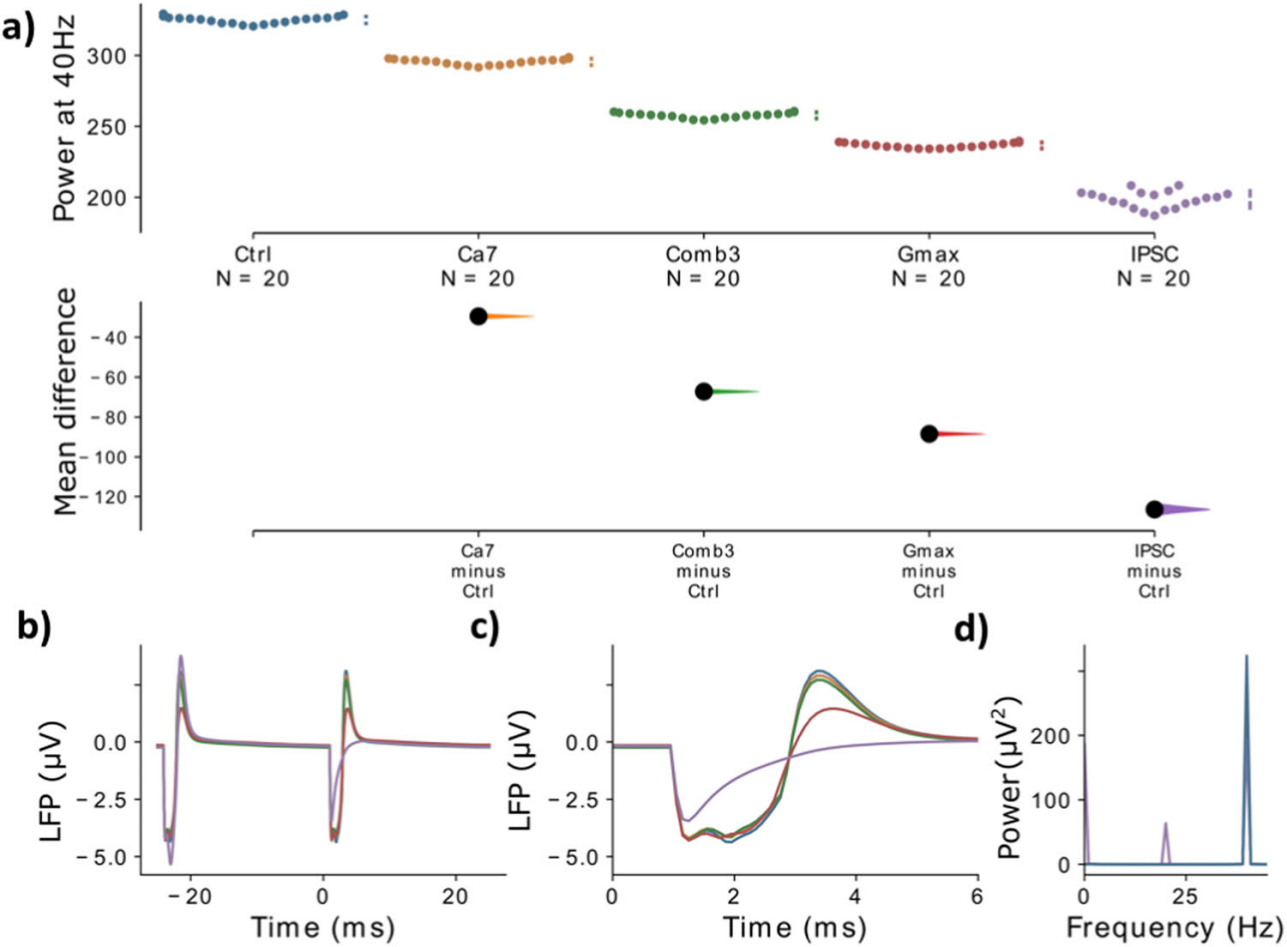
Comparison of model variants against synaptic alterations. The mean difference for the comparisons of the single model variant and the model variant combination with the strongest gamma reduction together with the two synaptic mechanisms, *Gmax* and *IPSC*, against the shared control are shown in the above Cumming estimation plot. The raw data is plotted on the upper axes. On the lower axes, mean differences are plotted as bootstrap sampling distributions. Each mean difference is depicted as a dot. Each 95% confidence interval is indicated by the ends of the vertical error bars. **b** The simulated LFP signal for the control network (blue), Ca7 (yellow), combination 3 (green), Gmax (red) and IPSC (purple) is shown, averaged over two consecutive gamma cycles. **c** The signals from **b** are presented with a zoom into the narrow time frame after the stimulus arrives at 0 ms. **d** The power spectral density (PSD) for the LFP signals from **b**. Note that in **b**–**d** the LFP signal is first averaged over all ‘subjects’ and ‘trials’.

Figure 5 shows that, while even the strongest individual model variants only result in moderate reductions of gamma power, combinations of several model variants can produce strong gamma reduction comparable to a strong reduction of GABA levels (see also Supplementary Table 4). Interestingly, the increase in GABAergic decay times produced a substantially stronger reduction of gamma power, mainly due to the emergence of a beat-skipping behavior, which shifted the power from the 40 Hz gamma band to the subharmonic 20 Hz beta band (see Supplementary Fig. 2). Nevertheless, these results demonstrate that combinations of model variants, influencing ionic channels and single cell excitability, can potentially have strong effects on gamma entrainment.

### Genetic variants do not affect the inter-trial phase coherence

**Fig. 6:**
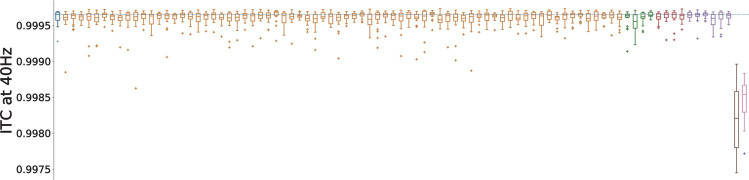
Overview of 4040 inter-trial coherence for all variants. Control network in blue, blue dashed line represents the mean of the control group, *Ca*^2+^ channel model variants affecting Ca_*HVA*_ in yellow, *Ca*^2+^ channel model variants affecting Ca_*LVA*_ in green, HCN model variants in red, SCN model variants in purple, the reduction of *g*_*max*_ in brown and increased IPSC times in steel pink. For each color-coded group of variants the ordering matches the ordering in Supplementary Tables 3, 4 and 5, respectively. Solid line represents the mean, box edges the 25 and 75 percentile, respectively, the whisker extend to 2 standard deviations and + depict outliers. the dashed blue line depicts the mean of the control network.

Besides strong reductions of evoked power, many studies report a decrease in inter-trial phase coherence (ITC), a measure of how aligned the phase angles of the signal over individual trials are, during gamma entrainment in patients with schizophrenia (e.g. refs. ^21,23^, see Thune et al.^24^ for a review). Therefore, we also calculated the ITC for each single model variant and compared them to the two synaptic conditions from before (see Methods section). We found that none of the model variants altered the phase coherence substantially, while both synaptic conditions strongly decreased ITC (Fig. 6). This means that while both synaptic conditions reduce phase coherence, i.e. desynchronize the network, the reduction in gamma power resulting from the model variants seems to come solely from a reduction in amplitude of the entrained oscillations, leaving the temporal precision intact. Furthermore, this suggests that, while genetic variants of ion channel-encoding genes might contribute to the reductions in evoked gamma power found in patients with schizophrenia, it is unlikely that they play an important role in the emergence of decreases in phase coherence and the underlying desynchronization of activity in the network.

### Correlations to pre-pulse inhibition and delta resonance

Lastly, we compared the effect of the genetic variants with their effect on other potential biomarkers for SCZ from our earlier studies^10^. Specifically, we compare the ratio of gamma reduction for each model variant from our study with the resonance power in the delta band and the pre-pulse inhibition (PPI) thresholds from the model described in ref. ^10^. We want to emphasize that this model and the model presented here differ in some important aspects, most importantly the model from our previous work is stimulated with amplitude-modulated noise and does not model ASSR click-train input. Nevertheless, both models are based on the same model of layer 5 pyramidal cells and implemented the same model variants with exactly the same procedure, making the results comparable. More details can be found in the Supplementary Methods 1.1.5. We calculated Pearson correlation coefficients between the ratio of gamma reduction and the resonance power in the delta band and the pre-pulse inhibition (PPI) thresholds for all 86 model variants. We found a strong positive correlation between gamma power and resonance power in the delta band (Pearson *r* = 0.683, *p* < 0.0001) and moderate negative correlation between gamma power and PPI threshold (Pearson *r* = −0.365, *p* < 0.0001) (see also Supplementary Figs. 4 and 5, respectively).

## Discussion

In this modeling study we showed that changes to the kinetics of voltage-gated ion channels due to SCZ-associated common variants of their encoding genes led to decreases of evoked gamma power, one of the most frequently reported electrophysiological phenotypes in SCZ (Figs. 2 and 3). We further demonstrated that combinations of model variants produced larger decreases in evoked gamma power (Fig. 4) and that these decreases were comparable to alterations at the synaptic level (Fig. 5), which are more commonly associated with changes of evoked oscillatory power in the gamma range^22,34^. This finding is in line with the notion that in a highly polygenic disorder such as schizophrenia single SNP variations only affect the phenotype very subtly and that the coincidence of several, presumably many, of these variations is necessary so that a clinical phenotype manifests. Furthermore, we did find that while specific combinations of model variants yielded larger decreases in evoked gamma power as stated above, for some other combinations, with opposed changes to the ion channel dynamics, the effects canceled out producing only negligible changes to the evoked gamma power (data not shown). Overall, we saw a continuum of decreases of evoked power for different combinations in line with the heterogeneity seen in patients with schizophrenia. Interestingly, we found that the genetic variants, opposed to the synaptic alterations, did not change the inter-trial phase coherence, a measure of synchronization. The reductions in evoked gamma power were solely due to reductions in the amplitude of the oscillations (Fig. 5).

The genetic variants that decreased evoked gamma power identified in this study mostly decreased the excitability of the layer 5 pyramidal cell, as found in earlier work^8^, and, therefore, led to smaller amplitudes of the evoked LFP signals. Not surprisingly, there was not much overlap with the variants we previously found to substantially increase network delta oscillations and to reduce single cell PPI^10^. We further analyzed this by correlating gamma power with resonance power in the delta band and PPI threshold from this previous study, respectively. Here, we found a strong positive correlation between gamma and resonance power in the delta band and a moderate negative correlation with PPI thresholds. This suggests that variants decreasing gamma power also lead to lower resonance power in the delta band and larger PPI thresholds. While robust evidence for decreased PPI thresholds in SCZ exists^4^, the findings on delta power show mixed results. Several studies find increased delta power in patients^46–50^, however, decreased delta oscillation power has also been reported^51,52^. Nonetheless, as also described earlier, it seems unlikely that gamma power reduction, delta power changes and PPI threshold decrease are solely caused by genetic variants affecting ionic channels. Gamma reductions most certainly are at least partially attributable to synaptic alterations as explained earlier. Nevertheless, the variants modeled here might play an important role in gamma reduction in subpopulations of patients and contribute to the large heterogeneity observed in patients with schizophrenia.

While our model consisting of populations of multi-compartment Hodgkin-Huxley type neurons is too complex to derive an analytical understanding of the effect of the genetic variants on gamma ASSRs, the current model could, in principle, be simplified to reveal such insight. For example, the layer 5 pyramidal cell model together with the effect of genetic variants on specific ion channels could be reduced to an adaptive exponential integrate fire model following the procedures in ref. ^53^ and the resulting network of these neurons could then further be analyzed using mean-field approaches detailed in ref. ^54^ and in ref. ^55^.

Previous models of gamma range oscillatory deficits in SCZ have mainly focused on changes at the synaptic level, such as changes of GABAergic synapses from PV^+^ interneurons onto pyramidal cells or other PV^+^ interneurons^22,34,35,37,45,56^, changes of glutamatergic excitation of PV^+^ interneurons through NMDA receptors,^37,57^ or changes of spine density at pyramidal cells^37^. As mentioned in the Results section, there are two main consequences of changes to the GABAergic system that have been the focus of previous modeling studies: 1) A reduction of the peak amplitude of the IPSC and 2) a prolongation of IPSC decay times. A reduction of the peak IPSC amplitude has been shown to significantly reduce evoked gamma power but to leave beta power intact^34,37,45^. On the other hand, an increase of IPSC decay time, while also substantially reducing evoked gamma power, has been shown to increase power in the beta band^22,34^ and most probably exerts its effect through PV^+^ basket cells^35^. Kirli et al.^57,58^ demonstrated that lower gamma band power was present at both low and high NMDA conductance levels with optimal synchronization occurring at intermediate conductance levels. In another study, Siekmeier and van Maanen^37^ showed that modest reductions in NMDA system function and dendritic spine density led to a robust reduction of gamma power. However, they also found that greater NMDA hypofunction along with low level GABA system dysregulation substantially decreased gamma power, highlighting the multifactoriality of underlying alterations. In addition, dopaminergic modulation of local circuits has also been shown to affect gamma synchronization through modulation of K^+^ currents and NMDA conductances^36^. Overall we must note that, as demonstrated for models of gamma ASSRs in particular^34,37^ but also other models of psychiatric disorders^59^ and models of healthy local circuits^60^ in general, many different parameter combinations might produce similar network level behavior. Therefore, it is very important to explore the interaction of alterations of different systems. Moreover, to constrain the models further, these interactions should be tested against different paradigms, such as spontaneous and evoked oscillations. The computational model presented here offers an ideal starting point for such an effort, since it allows for the integration of the most crucial factors contributing to gamma band oscillatory deficits in schizophrenia. Beyond incorporating variants of ion channel-encoding genes and alterations of GABAergic synapses, extensions of the model could include the integration of NMDAR hypofunction via its NMDARs and the integration of changes to dopaminergic neuromodulation via its K^+^ channels, for example as in ref. ^36^.

While there is strong evidence that PV^+^ inhibitory interneurons play a very important role in the generation and maintenance of local cortical gamma oscillations^32,33^, other inhibitory interneuron populations might also be involved. For instance, Veit et al.^61^ demonstrated that context-dependent gamma rhythms critically depend on dendrite-targeting, somatostatin-positive (SST^+^) interneurons in the primary visual cortex of mice. Moreover, Veit et al.^62^ very recently showed that also vasoactive intestinal peptide-positive (VIP^+^) neurons can suppress spectral coherence between distal cortical ensembles during the processing of non-matching stimulus properties. Interestingly, cellular, molecular and synaptic changes to SST^+^ interneurons have also been found in patients with schizophrenia. For example, Hashimoto et al.^63^ found a reduced expression of SST^+^, GAD67 and GAT1 in cortical inhibitory interneurons and, interestingly, significantly correlated expression changes of SST^+^ and GAD67, but not of SST^+^ and GAT1. Furthermore, Morris et al.^64^ observed that both the density of SST^+^ neurons and the expression of SST^+^ per neuron was reduced in SCZ. These changes have been found in most cortical layers with varying strength^64,65^ and can be observed throughout cortex^66^. Subsequently, these changes to SST^+^ interneurons might also contribute to the gamma oscillation deficits seen in schizophrenia and our minimalistic model, consisting only of pyramidal cells and PV^+^ interneurons might not capture the full nature of SCZ-associated changes. However, we omitted including more interneuron subtypes for the following reasons: 1) SST^+^ and VIP^+^ interneurons involvement in gamma oscillations as described above seems to be mainly involved in gamma coherence between distal cortical ensembles during the processing of different aspects of the stimulus and not in the local generation of the gamma oscillations in cortical microcolumns. This synchronization likely plays a minor role in ASSR paradigms where the cortical network is passively driven by a simple periodic stimulus. 2) While there is evidence for changes to SST^+^ interneurons in patients with SCZ, as outlined above, the changes to PV^+^ interneurons seem to be more grave and more severely alter their influence over the network activity. 3) Tuning such a detailed model requires optimizing free parameters such as connectivity weights and the addition of further cell populations increases the number of free parameters (growing with *n*^2^ for the number *n* of cell populations), making the tuning processes much more expensive. Therefore, we decided to restrict our analysis to a minimal model for the generation of gamma oscillations in terms of represented cell populations. However, we do acknowledge that, for example building on the work presented here or even more realistic models such as the one presented in Dura-Bernal et al.^67^, an exploration of the influence of other interneuron subtypes on the gamma auditory steady-state response is warranted.

A crucial part of the overall approach used in this study is the dampening of the effect of literature-derived model variants by a downscaling of the parameter changes. Typically, the literature-derived model variants very strongly changed the physiology of the studied cell (see also ref. ^8^) and, therefore, were not representative of common SNP-like variants. Overall, single SNP-like variants, which are known to be numerous and to occur frequently in the healthy population^38^, are assumed to have small phenotypic effects, either on the single cell or on the systems level. Nevertheless, we note that rare variants with large effects associated with schizophrenia also exist (see e.g. ref. ^68^). Additionally, we have shown in previous work on increased delta oscillations due to genetic variants that model variants derived from gene expression data largely result in similar changes to the network model^10^.

Genome-wide association studies (GWAS) studies, such as Ripke et al.^38^, have identified numerous variants associated with psychiatric disorders, however, we know very little about their functional effects. As we have argued before^9,10^, the modeling framework presented in this study is ideally suited to build hypotheses about their effects and to make experimentally testable predictions. To be more specific, the biophysically detailed model used here can provide very specific associations between genetic variants and phenotypes, while explicitly revealing the cellular properties through which the two are mechanistically linked. This goes well beyond the purely statistical associations that standard genetics approaches produce.

The analysis presented here is based on the model of a thick-tufted layer 5 pyramidal cell, which accurately reproduces many active and passive electrophysiological features of these cells^69^, and the effects of the genetic variants were implemented as changes to the kinetics of the underlying ion channels of the model. Therefore, our approach here rests on the assumption that the model faithfully reproduces the ion channel dynamics of layer 5 pyramidal cells. While the fitting of the model did not include the replication of activity in the presence of ion-channel blockers^70^, the model’s ion channel composition is largely consistent with that of other models. Almog et al. present a model of a layer 5 pyramidal cell where the set of channels is partly overlapping^71^. Although the contributions of the ion channels to model behavior differ slightly, with the persistent K^+^ having a larger role, the two models mostly conform with each other^71,72^. Papoutsi et al.^73^ also present a model of layer 5 pyramidal cells showing similar interactions between voltage-gated Ca^2+^ channels and the Ca^2+^-dependent afterhyperpolarization (AHP) current as in the model of Hay et al. we used here and further underpin the validity of our model assumptions.

While in this study we have focused on the effect of genetic variants on ion channel dynamics, future studies should also explore their role in schizophrenia-associated changes to synaptic receptors, especially GABA and NMDA receptors. These are, as mentioned before, more traditionally associated with gamma band deficits in the disorder^32,74^. Furthermore, the approach outlined in this study could also be extended to study the effect of genetic variants on intracellular signaling cascades involved in plasticity^39,75^. A major challenge for the field, however, will be to incorporate immune pathways, which have been strongly indicated by recent GWASs^38,76^, into models of schizophrenia pathophysiology.

Nevertheless, the model developed in this study can already be used to complement more traditional approaches to identify potential treatment targets. Current medications with known courses of action can be included into the model and their effects on gamma band oscillations can subsequently be assessed (see ref. ^37^ for an excellent implementation of such an approach). Furthermore, a search for novel therapeutic targets can be conducted through a more exploratory analysis of the effect of parameters on the model behavior (also see ref.^37^). Additionally, such an approach is not limited to pharmacological interventions since transcranial electric or magnetic stimulation can easily be incorporated (as demonstrated in other modeling studies such as ref. ^77–79^); this is, of course, not restricted to schizophrenia but can be applied to psychiatric disorders in general.

In conclusion, our work represents a step towards the integration of the wealth of genetic data on psychiatric disorders into biophysically detailed models of biomarkers with great potential to unravel underlying polygenic cellular-based mechanisms. Furthermore, the approach offers an ideal test ground for the identification of novel therapeutic strategies, such as pharmaceutical interventions or electrical stimulation.

## Methods

### Network model

**Fig. 7:**
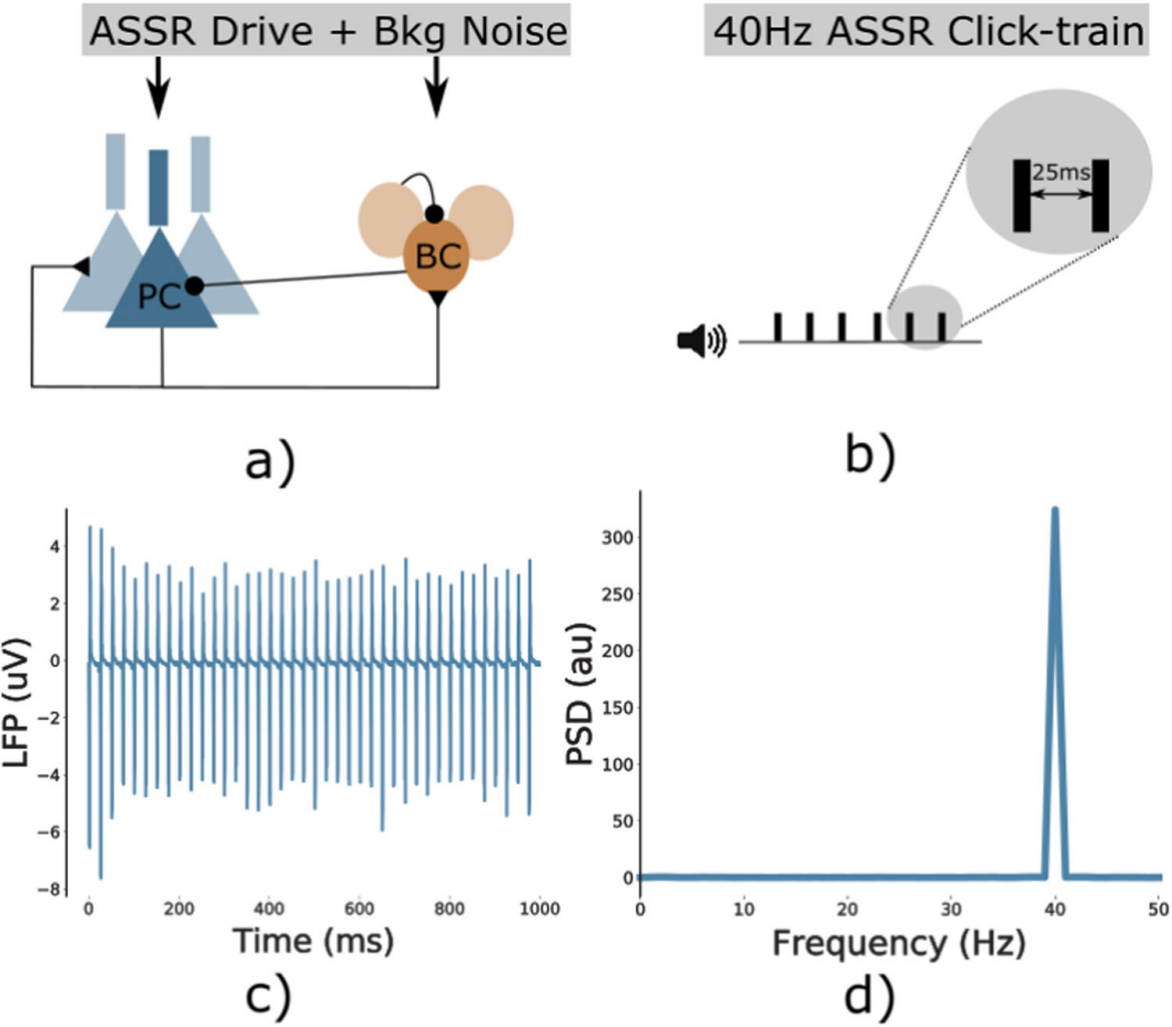
Overview. **a** Network schematic. The network consists of two interconnected populations, an excitatory population of pyramidal cells and an inhibitory population of basket cells, both receiving rhythmic ASSR drive and Poissonian background noise. **b** The 40 Hz ASSR drive is modeled as bouts of input spikes arriving at all cells simultaneously with an inter-bout interval of 25 ms, mimicking a 40 Hz ASSR click-train paradigm. **c** Example LFP signal of the control network in response to the ASSR drive, showing strong 40 Hz entrainment. **d** Power spectral density of the signal from **c**, again confirming that the network follows the 40 Hz click train rhythm.

This study was based on a high-complexity, biophysically detailed model of thick-tufted layer 5 pyramidal cells^69^. The model includes a detailed reconstructed morphology, models of the dynamics of eleven different ionic channels and a description of the intracellular Ca^2+^ concentration^69^. Following earlier work, we incorporated human in vitro electrophysiological data on ion channel behavior from the functional genomics literature into this model^8,10^. Due to the computational complexity of the original model, consisting of 196 compartments, we decided to use a reduced-morphology model, where passive parameters, ion channel conductances and parameters describing Ca^2+^ dynamics were fitted to reproduce the behavior of the original model^40^. The inhibitory cells in the network were based on a model of fast-spiking PV^+^ basket cells taken from ref. ^41^. These two single cell models were combined into a microcircuit network model consisting of 256 excitatory and 64 inhibitory neurons (Fig. 7). Cells were connected via AMPA and NMDA receptor-mediated synaptic currents in the case of excitatory connections and GABA_*A*_ receptor-mediated synaptic currents for inhibitory connections. Additionally, model cells received two types of input, Poissonian noise to all cells representing background activity in the cortex and rhythmic input representing the sensory input during auditory entrainment. Note that a smaller percentage of inhibitory interneurons (35%) received no sensory input drive; this reflects preferential thalamic drive to pyramidal cell populations^80^, which has also been used in other models^22^. This was to ensure that a subpopulation of the inhibitory neurons had a weak enough drive to be dominated by pyramidal cell activity. This subpopulation was necessary to maintain a 20 Hz peak for 40 Hz drive in of the synaptic alteration conditions against which we compared the genetic alterations. For a detailed discussion see the study by Vierling-Claassen et al.^22^.

For more information on the network model and data analysis, see the Supplementary Material.

### Integration of genetic variants

We closely follow earlier work of refs. ^8,10,72^ to integrate the effect of SNP-like genetic variants into our network model (details in Supplementary Section 1.1.3). In summary, we selected a set of genes, restricted to ion-channel-encoding genes likely to be expressed in layer 5 pyramidal cells, obtained from a large GWAS^38^. For this set of genes, we searched the literature for genetic variants and their effects on electrophysiological parameters of pyramidal cells. This left us with 86 variants of the following genes: CACNA1C, CACNA1D, CACNB2, SCN1A and, HCN1^81–104^. These variants typically had a large effect on the electrophysiology of layer 5 pyramidal cells. However, due to the polygenic nature of SCZ, it can be assumed that the risk of the disorder is not caused by a single SCZ-associated SNP and, therefore, one would not expect large effects from the common variants identified in GWAS. Subsequently, we applied a downscaling procedure, as outlined in refs. ^8,10^. In short, we downscaled the changes of model parameters induced by a variant (multiplication by a factor either on a linear or logarithmic scale, depending on the type of the parameter) until the cell response to predefined stimuli stayed within a certain range (Supplementary Section 1.1.3). This resulted in a set of 86 ’small-effect’ model variants, which were used as models for the effects of common variants on layer 5 pyramidal cell electrophysiological response features. As in earlier studies using this approach^8,10^, we will use the term ’variant’ for a genetic variant in a human or animal genome and the term ’model variant’ for a model of a gene variant constructed as described above.

## Supplementary information

Supplementary Material

## Data Availability

All code to simulate the computational model and generate the data analyzed in the study together with processed generated data is available at https://github.com/ChristophMetzner/ACnet.

## Code availability

All code to simulate the computational model and generate the data analyzed in the study, to analyze the data and to create the figures is available at https://github.com/ChristophMetzner/ACnet/). The model code will also be made available through ModelDB (https://senselab.med.yale.edu/modeldb/) upon publication. Furthermore, the model will also be included in the ASSRUnit package, which is designed for automated validation and comparison of models of ASSR deficits in psychiatric disorders^105^.
