# Supplementary material for: The effect of alterations of schizophrenia-associated genes on gamma band oscillations
Source: Schizophrenia (Heidelb). 2022 Apr 28;8(1):46. doi: 10.1038/s41537-022-00255-7 (PMC9261091; doi:10.1038/s41537-022-00255-7)
Supplement: Supplementary file 1 — Supplementary Material [file 41537_2022_255_MOESM1_ESM.pdf]

The Effect of Alterations of Schizophrenia-Associated Genes on Gamma Band Oscillations - Supplementary Material

Christoph Metzner[1,2], Tuomo Mäki-Marttunen[3], Gili Karni[1,4], Hana McMahon-Cole[1,4], and Volker Steuber[2]

[1] *Neural Information Processing Group, Institute of Software Engineering and Theoretical Computer Science, Technische Universität Berlin, Berlin, Germany*

[2] *Biocomputation Research Group, School of Physics, Engineering and Computer Science, University of Hertfordshire, Hatfield, United Kingdom*

[3] *Department of Computational Physiology, Simula Research Laboratory, Oslo, Norway*

[4] *Minerva Schools at KGI, San Francisco, USA*

March 31, 2022

1 Supplementary Material

1.1 Supplementary Methods

Ion channels and their genetic etiology

The single cell model used to model the pyramidal cells in our network is based on a detailed, multi-compartment Hodgkin-Huxley type model of layer 5 pyramidal cells with a reconstructed morphology [16]. However, the very high morphological detail (196 compartments), and thus very high computational complexity, of this neuron model renders it unsuitable for the network model analysis performed in this study. Therefore, we employed a reduced version of this model, where passive parameters and ion channel and Ca^{2+} dynamics were fitted to the original model using a multi-step fitting procedure [32]. The reduced model, analogous to the original model, contains the following ionic currents: Fast inactivating Na^{+} current (I_{Nat}), persistent Na^{+} current (I_{Nap}), non-specific cation current (I_{h}), muscarinic K^{+} current (I_{m}), slow inactivating K^{+} current (I_{Kp}), fast inactivating K^{+} current (I_{Kt}), fast non-inactivating K^{+} current ($I_{\mathrm{Kv3.1}}$), high-voltage-activated Ca^{2+} current (I_{CaHVA}), low-voltage-activated Ca^{2+} current (I_{CaLVA}), small-conductance Ca^{2+}-activated K^{+} current (I_{SK}), and the passive leak current (I_{leak}).

Exact information on which ion channel subunits contribute to the above mentioned currents in layer 5 pyramidal cells is still missing. However, the expression of different ion channel subunits in these cells has been studied extensively, and indications can be drawn from these studies. Christophe et al. [7] found the mRNA of

the ion channel-encoding genes *KCNA2*, *KCND2*, *KCND3*, *CACNA1A*, *CACNA1B*, *CACNA1C*, *CACNA1D*, *CACNA1E*, *CACNA1G*, *CACNA1H*, *CACNA1I*, *HCN1*, and *HCN2* expressed in postnatal rat neocortices at different stages of development. Of these genes, it is known that*CACNA1A*, *CACNA1B*, *CACNA1C*, and *CACNA1D* contribute to I_{CaHVA}, *CACNA1G*, *CACNA1H*, and *CACNA1I* to I_{CaLVA}, while the genes *KCNA2*, *KCND2*, and *KCND3* might contribute to the slow I_{Kp} current.

In a different study, the genes *SCN1A*, *SCN2A*, *SCN3A*, and *SCN6A* were found to be expressed in layer 5 pyramidal cells [58]. While several of the genes encoding the α subunits (SCN1A, SCN2A and SCN3A) are tetrodotoxin-sensitive [4] and hence form both the transient (I_{Nat}) and persistent (I_{Nap}) Na^+ currents, their contribution to I_{Nat} or I_{Nap} might be dependent on modulatory subunits [29].

1.1.1 Description of the Single Cell Models

Layer 5 pyramidal cells The layer 5 pyramidal cell model is a multi-compartment Hodgkin-Huxley type model and the membrane potential dynamics can be described by

$$C_m \frac{\partial V}{\partial t} = I_{\text{Nat}} + I_{\text{Nap}} + I_h + I_m + I_{\text{Kp}} + I_{\text{Kt}} + I_{\text{Kv3.1}} + I_{\text{CaHVA}} + I_{\text{CaLVA}} + I_{\text{SK}} + I_l + I_{axial},$$

where the different currents can be written as the product of an activation and an inactivation variable

$$I = \bar{g} m^{N_m} h^{N_h} (E - V).$$

Here, m and h are the activation and inactivation variables, N_m and N_h their sensitivities, $\bar{g}$ the maximal ionic conductance and E the ionic reversal potential. Na^+ and

K^+ have a fixed reversal potential of $E_{Na} = 50\,\mathrm{mV}$ and $E_{Na} = -85\,\mathrm{mV}$, respectively, while the reversal potential of Ca^{2+} depends on the intracellular $[Ca^{2+}]$. Furthermore, the dynamics of activation and inactivation are defined by

$$\frac{dm}{dt} = \frac{m - m_\infty}{\tau_m} \qquad \text{and} \qquad \frac{dh}{dt} = \frac{h - h_\infty}{\tau_h},$$

where m_∞, h_∞, τ_m, and τ_h are functions of the membrane potential V. In our case, m_∞ and h_∞ usually have a sigmoidal shape, where the half-activation and half-inactivation voltages are determined by one or more parameters each (depending on the ion channel). These parameters are written as $V_{\mathrm{offm}*}$ and $V_{\mathrm{offh}*}$, where $*$ stands for further specifications in case there are multiple parameters affecting them. Analogously, the parameters $V_{\mathrm{slom}*}$ and $V_{\mathrm{sloh}*}$ affect the slopes of the (in)activation curves, and parameters $\tau_{\mathrm{m}*}$ and $\tau_{\mathrm{offh}*}$ the time constants. All ionic currents, except for the activation of I_{SK}, are described in this way. The activation of I_{SK}, on the other hand, only depends on the intracellular $[Ca^{2+}]$, through a sigmoidal function with a half-activation concentration parameter c_{off} and a slope parameter c_{slo}. Lastly, the dynamics of the intracellular $[Ca^{2+}]$ is described by

$$\frac{d[Ca^{2+}]_i}{dt} = \frac{I_{\mathrm{CaHVA}} + I_{\mathrm{CaLVA}}}{2\gamma F d} - \frac{[Ca^{2+}]_i - c_{\mathrm{min}}}{\tau_{\mathrm{decay}}}, \tag{1}$$

where I_{CaHVA} and I_{CaLVA} are the high and low-voltage activated Ca^{2+} currents entering the considered cell segment, γ represents the fraction of Ca^{2+} ions entering the cell that contribute to the intracellular $[Ca^{2+}]$, F the Faraday constant, d is the depth of the sub-membrane layer considered for calculation of concentration, c_{min} the resting intracellular $[Ca^{2+}]$, and τ_{decay} is the decay time constant of the intracellular $[Ca^{2+}]$.

As mentioned earlier, in this study we employed the reduced model presented in [32], consisting of four compartments: the soma, the apical trunk, the apical tuft, and the basal dendrite. the maximal conductance values $\bar{g}$ were different for different compartments and are subindexed as follows: s refers to the soma, a_0 to the apical trunk, a_1 to the apical tuft, and b to the basal dendrite. All values not shown are set to 0.

Fast inactivating Na$^+$ current, I_{Nat}

$$\alpha_m = -\frac{1}{\tau_{\mathrm{ma}}} \cdot \frac{V_{\mathrm{offm}} - V}{1 - \exp(\frac{(V_{\mathrm{offm}}-V)}{V_{\mathrm{slom}}})}$$

$$\beta_m = \frac{1}{\tau_{\mathrm{mb}}} \cdot \frac{V_{\mathrm{offm}} - V}{1 - \exp(\frac{-(V_{\mathrm{offm}}-V)}{V_{\mathrm{slom}}})}$$

$$\alpha_h = \frac{1}{\tau_{\mathrm{ha}}} \cdot \frac{V_{\mathrm{offh}} - V}{1 - \exp(\frac{(V_{\mathrm{offh}}-V)}{V_{\mathrm{sloh}}})}$$

$$\beta_h = -\frac{1}{\tau_{\mathrm{hb}}} \cdot \frac{V_{\mathrm{offh}} - V}{1 - \exp(\frac{-(V_{\mathrm{offh}}-V)}{V_{\mathrm{sloh}}})}$$

$$m_\infty = \frac{\alpha_m}{\alpha_m + \beta_m}$$

$$h_\infty = \frac{\alpha_h}{\alpha_h + \beta_h}$$

$$\tau_m = \frac{1}{T_{\mathrm{adj}}(\alpha_m + \beta_m)}$$

$$\tau_h = \frac{1}{T_{\mathrm{adj}}(\alpha_h + \beta_h)}$$

$V_{\mathrm{offm}} = -38$ mV, $V_{\mathrm{offh}} = -66$ mV, $V_{\mathrm{slom}} = 6.0$ mV, $V_{\mathrm{sloh}} = 6.0$ mV, $\tau_{\mathrm{ma}} = 5.49$ ms, $\tau_{\mathrm{mb}} = 8.06$ ms, $\tau_{\mathrm{ha}} = 66.67$ ms, $\tau_{\mathrm{hb}} = 66.67$ ms, $\bar{g}_s = 2.41$ S/cm^2, $\bar{g}_{a_0} = 0.0135$ S/cm^2, $\bar{g}_{a_1} = 0.0131$ S/cm^2, $N_m = 3$, $N_h = 1$

Persistent Na$^+$ current, I_{Nap}

$$m_\infty = \frac{1}{1+\exp(\frac{V_{\mathrm{offm}}-V}{V_{\mathrm{slom}}})}$$

$$h_\infty = \frac{1}{1+\exp(-\frac{V_{\mathrm{offh}}-V}{V_{\mathrm{sloh}}})}$$

$$\alpha_m = -\frac{1}{\tau_{\mathrm{ma}}}\cdot\frac{V_{\mathrm{offma}}-V}{1-\exp(\frac{(V_{\mathrm{offma}}-V)}{V_{\mathrm{sloma}}})}$$

$$\beta_m = \frac{1}{\tau_{\mathrm{mb}}}\cdot\frac{V_{\mathrm{offmb}}-V}{1-\exp(\frac{-(V_{\mathrm{offmb}}-V)}{V_{\mathrm{slomb}}})}$$

$$\alpha_h = \frac{1}{\tau_{\mathrm{ha}}}\cdot\frac{V_{\mathrm{offha}}-V}{1-\exp(-\frac{V_{\mathrm{offha}}-V}{V_{\mathrm{sloha}}})}$$

$$\beta_h = -\frac{1}{\tau_{\mathrm{hb}}}\cdot\frac{V_{\mathrm{offhb}}-V}{1-\exp(\frac{V_{\mathrm{offhb}}-V}{V_{\mathrm{slohb}}})}$$

$$\tau_m = \frac{6}{T_{\mathrm{adj}}(\alpha_m+\beta_m)}$$

$$\tau_h = \frac{1}{T_{\mathrm{adj}}(\alpha_h+\beta_h)}$$

$V_{\mathrm{offm}} = -52.6$ mV, $V_{\mathrm{slom}} = 4.6$ mV, $V_{\mathrm{offma}} = -38$ mV, $V_{\mathrm{offmb}} = -38$ mV, $V_{\mathrm{sloma}} = 6.0$ mV, $V_{\mathrm{slomb}} = 6.0$ mV, $\tau_{\mathrm{ma}} = 5.49$ ms, $\tau_{\mathrm{mb}} = 8.06$ ms, $V_{\mathrm{offh}} = -48.8$ mV, $V_{\mathrm{sloh}} = 10.0$ mV, $V_{\mathrm{offha}} = -17$ mV, $V_{\mathrm{offhb}} = -64.4$ mV, $V_{\mathrm{sloha}} = 4.63$ mV, $V_{\mathrm{slohb}} = 2.63$ mV, $\tau_{\mathrm{ha}} = 347222.2$ ms, $\tau_{\mathrm{hb}} = 144092.2$ ms, $\bar{g}_s = 0.00206$ S/cm^2, $N_m = 3$, $N_h = 1$

Non-specific cation current, I_{h}

$$\alpha_h = -\frac{1}{\tau_{\mathrm{ha}}}\cdot\frac{V_{\mathrm{offha}}-V}{\exp(-\frac{V_{\mathrm{offha}}-V}{V_{\mathrm{sloha}}})-1}$$

$$\beta_h = \frac{1}{\tau_{\mathrm{hb}}}\exp(-\frac{V_{\mathrm{offhb}}-V}{V_{\mathrm{slohb}}})$$

$$h_\infty = \frac{\alpha_h}{\alpha_h + \beta_h}$$
$$\tau_h = \frac{1}{\alpha_h + \beta_h}$$

$E = -45.0$ mV, $V_{\text{offha}} = -154.9$ mV, $V_{\text{sloha}} = 11.9$ mV, $\tau_{\text{ha}} = 155.52$ ms, $V_{\text{offhb}} = 0.0$ mV, $V_{\text{slohb}} = 33.1$ mV, $\tau_{\text{hb}} = 5.18$ ms, $\bar{g}_s = 0.000279$ S/cm^2, $\bar{g}_{a_1} = 0.00493$ S/cm^2, $\bar{g}_b = 0.000294$ S/cm^2, $N_m = 0$, $N_1 = 0$

Muscarinic K$^+$ current, I_{m}

$$\alpha_m = \frac{1}{\tau_{\text{ma}}} \exp\left(-\frac{V_{\text{offma}} - V}{V_{\text{sloma}}}\right)$$
$$\beta_m = \frac{1}{\tau_{\text{mb}}} \exp\left(\frac{V_{\text{offmb}} - V}{V_{\text{slomb}}}\right)$$
$$m_\infty = \frac{\alpha_m}{\alpha_m + \beta_m}$$
$$\tau_m = \frac{1}{T_{\text{adj}}(\alpha_m + \beta_m)}$$

$V_{\text{offma}} = -35$ mV, $V_{\text{sloma}} = 10$ mV, $\tau_{\text{ma}} = 303.03$ ms, $V_{\text{offmb}} = -35$ mV, $V_{\text{slomb}} = 10$ mV, $\tau_{\text{mb}} = 303.03$ ms, $\bar{g}_{a_0} = 0.000143$ S/cm^2, $\bar{g}_{a_1} = 0.000113$ S/cm^2, $N_m = 1$, $N_h = 0$

Slow inactivating K$^+$ current, I_{Kp}

$$m_\infty = \frac{1}{1 + \exp\left(\frac{V_{\text{offm}} - V}{V_{\text{slom}}}\right)}$$
$$h_\infty = \frac{1}{1 + \exp\left(-\frac{V_{\text{offh}} - V}{V_{\text{sloh}}}\right)}$$
$$\tau_m = \begin{cases} \frac{\tau_{\text{mmin}} + \tau_{\text{mdiff1}} \exp\left(-\frac{V_{\text{offmt}} - V}{V_{\text{slomt}}}\right)}{T_{\text{adj}}}, & \text{if } V \leq V_{thresh} \\ \frac{\tau_{\text{mmin}} + \tau_{\text{mdiff2}} \exp\left(\frac{V_{\text{offmt}} - V}{V_{\text{slomt}}}\right)}{T_{\text{adj}}}, & \text{if } V > V_{thresh} \end{cases}$$

$$\tau_h = \frac{\tau_{\text{hmin}} + (\tau_{\text{hdiff1}} - \tau_{\text{hdiff2}}(V_{\text{offht1}} - V))\exp\left(-\left(\frac{V_{\text{offht2}}-V}{V_{\text{sloht}}}\right)^2\right)}{T_{\text{adj}}}$$

$$V_{thresh} = V_{\text{offmt}} - \frac{V_{\text{slomt}}}{2}\log\left(\frac{\tau_{\text{mdiff1}}}{\tau_{\text{mdiff2}}}\right)$$

$V_{\text{offm}} = -11$ mV, $V_{\text{slom}} = 12$ mV, $V_{\text{offmt}} = -10$ mV, $V_{\text{slomt}} = 38.46$ mV, $\tau_{\text{mmin}} = 1.25$ ms, $\tau_{\text{mdiff1}} = 175.03$ ms, $\tau_{\text{mdiff2}} = 13$ ms, $V_{\text{offh}} = -64$ mV, $V_{\text{sloh}} = 11$ mV, $V_{\text{offht1}} = -65$ mV, $V_{\text{offht2}} = -85$ mV, $V_{\text{sloht}} = 48$ mV, $\tau_{\text{hmin}} = 360$ ms, $\tau_{\text{hdiff1}} = 1010$ ms, $\tau_{\text{hdiff2}} = 24$ ms/mV, $\bar{g}_s = 0.000176$ S/cm^2, $N_m = 2$, $N_h = 1$

Fast inactivating K$^+$ current, I_{Kt}

$$m_\infty = \frac{1}{1 + \exp(\frac{V_{\text{offm}}-V}{V_{\text{slom}}})}$$

$$h_\infty = \frac{1}{1 + \exp(-\frac{V_{\text{offh}}-V}{V_{\text{sloh}}})}$$

$$\tau_m = \frac{\tau_{\text{mmin}} + \tau_{\text{mdiff}}\exp\left(-\left(\frac{V_{\text{offmt}}-V}{V_{\text{slomt}}}\right)^2\right)}{T_{\text{adj}}}$$

$$\tau_h = \frac{\tau_{\text{hmin}} + \tau_{\text{hdiff}}\exp\left(-\left(\frac{V_{\text{offht}}-V}{V_{\text{sloht}}}\right)^2\right)}{T_{\text{adj}}}$$

$V_{\text{offm}} = -10$ mV, $V_{\text{slom}} = 19$ mV, $V_{\text{offh}} = -76$ mV, $V_{\text{sloh}} = 10$ mV, $V_{\text{offmt}} = -81$ mV, $V_{\text{slomt}} = 59$ mV, $\tau_{\text{mmin}} = 0.34$ ms, $\tau_{\text{mdiff}} = 0.92$ ms, $V_{\text{offht}} = -83$ mV, $V_{\text{sloht}} = 23$ mV, $\tau_{\text{hmin}} = 8$ ms, $\tau_{\text{hdiff}} = 49$ ms, $\bar{g}_s = 0.0239$ S/cm^2, $N_m = 4$, $N_h = 1$

Fast, non inactivating K$^+$ current, $I_{\text{Kv3.1}}$

$$m_\infty = \frac{1}{1+\exp(\frac{V_{\text{offm}}-V}{V_{\text{slom}}})}$$

$$h_\infty = \frac{1}{T_{\text{adj}}\left(1+\exp(\frac{V_{\text{offh}}-V}{V_{\text{sloh}}})\right)}$$

$V_{\text{offma}} = 18.7$ mV, $V_{\text{offmt}} = -46.56$ mV, $V_{\text{sloma}} = 9.7$ mV, $V_{\text{slomt}} = 44.14$ mV, $\tau_{\text{mmax}} = 4.0$ ms, $\bar{g}_s = 0.701$ S/cm^2, $\bar{g}_{a_0} = 0.00121$ S/cm^2, $N_m = 1$, $N_h = 0$

High-voltage-activated Ca^{2+} current, I_{CaHVA}

$$\alpha_m = -\frac{1}{\tau_{\text{ma}}} \cdot \frac{V_{\text{offma}} - V}{1-\exp(\frac{V_{\text{offma}}-V}{V_{\text{sloma}}})}$$

$$\beta_m = \frac{1}{\tau_{\text{mb}}} \exp(-\frac{V_{\text{offmb}} - V}{V_{\text{slomb}}})$$

$$m_\infty = \frac{\alpha_m}{\alpha_m + \beta_m}$$

$$\tau_m = \frac{1}{\alpha_m + \beta_m}$$

$$\alpha_h = \frac{1}{\tau_{\text{ha}}} \exp(\frac{V_{\text{offha}} - V}{V_{\text{sloha}}})$$

$$\beta_h = -\frac{1}{\tau_{\text{hb}}} \cdot \frac{1}{1+\exp(\frac{(V_{\text{offhb}}-V)}{V_{\text{slohb}}})}$$

$$h_\infty = \frac{\alpha_h}{\alpha_h + \beta_h}$$

$$\tau_h = \frac{1}{\alpha_h + \beta_h}$$

$V_{\text{offma}} = -27$ mV, $V_{\text{offmb}} = -75$ mV, $V_{\text{offha}} = -13$ mV, $V_{\text{offhb}} = -15$ mV, $V_{\text{sloma}} = 3.8$ mV, $V_{\text{slomb}} = 17$ mV, $V_{\text{sloha}} = 50$ mV, $V_{\text{slohb}} = 28$ mV, $\tau_{\text{ma}} = 18.18$ ms, $\tau_{\text{mb}} = 1.06$ ms, $\tau_{\text{ha}} = 2188.18$ ms, $\tau_{\text{hb}} = 153.85$ ms, $\bar{g}_s = 0.000838$ S/cm^2, $\bar{g}_{a_1} = 0.000977$ S/cm^2, $N_m = 2$, $N_h = 1$

Low-voltage-activated Ca^{2+} current, I_{CaLVA}

$$m_\infty = \frac{1}{1 + \exp(\frac{V_{\mathrm{offm}} - V}{V_{\mathrm{slom}}})}$$
$$h_\infty = \frac{1}{1 + \exp(-\frac{V_{\mathrm{offh}} - V}{V_{\mathrm{sloh}}})}$$

$$\tau_m = \tau_{\mathrm{mmin}} + \frac{\tau_{\mathrm{mdiff}}}{T_{\mathrm{adj}}\left(1 + \exp(-\frac{V_{\mathrm{offmt}} - V}{V_{\mathrm{slomt}}})\right)}$$
$$\tau_h = \tau_{\mathrm{hmin}} + \frac{\tau_{\mathrm{hdiff}}}{T_{\mathrm{adj}}\left(1 + \exp(-\frac{V_{\mathrm{offht}} - V}{V_{\mathrm{sloht}}})\right)}$$

$V_{\mathrm{offma}} = -40.0$ mV, $V_{\mathrm{offmt}} = -35.0$ mV, $V_{\mathrm{offha}} = -90.0$ mV, $V_{\mathrm{offht}} = -50.0$ mV, $V_{\mathrm{sloma}} = 6.0$ mV, $V_{\mathrm{slomt}} = 5.0$ mV, $V_{\mathrm{sloha}} = 6.4$ mV, $V_{\mathrm{sloht}} = 7.0$ mV, $\tau_{\mathrm{mmin}} = 5.0$ ms, $\tau_{\mathrm{mdiff}} = 20.0$ ms, $\tau_{\mathrm{hmin}} = 20.0$ ms, $\tau_{\mathrm{hdiff}} = 50.0$ ms, $\bar{g}_s = 0.00311$ S/cm^2, $\bar{g}_{a_1} = 0.000487$ S/cm^2, $N_m = 2$, $N_h = 1$

Small-conductance Ca^{2+}-activated K^+ current, I_{SK}

$$m_\infty = \frac{1}{1 + \left(\frac{[\mathrm{Ca}^{2+}]_i}{c_{\mathrm{off}}}\right)^{-c_{\mathrm{slo}}}}$$

$c_{\mathrm{off}} = 0.00043$ mM, $c_{\mathrm{slo}} = 4.8$, $\bar{g}_s = 0.0479$ S/cm^2, $\bar{g}_{a_0} = 0.000231$ S/cm^2, $\bar{g}_{a_1} = 0.00365$ S/cm^2, $N_m = 1$, $N_h = 1$

Leak current, I_{leak}

$E = -90$ mV, $\bar{g}_s = 0.000078$ S/cm^2, $\bar{g}_{a_0} = 0.0000592$ S/cm^2, $\bar{g}_{a_1} = 0.0000675$ S/cm^2,

$\bar{g}_b = 0.0000256$ S/cm^2, $N_m = 0$, $N_h = 0$

Intracellular [Ca^{2+}] dynamics

The intracellular Ca^{2+} concentration follows Equation 1 with the following model parameters: $\gamma_s = 0.0005$, $\gamma_{a_0} = 0.0347$, $\gamma_{a_1} = 0.0005$, $\tau_{\text{decay},s} = 488$ ms, $\tau_{\text{decay},a_0} = 142$ ms, $\tau_{\text{decay},a_1} = 95.4$ ms, $d = 0.1$ µm, $c_{\min} = 10^{-4}$ mM

Temperature adjustment factor: $T_{\text{adj}} = 2.3^{\frac{34-21}{10}}$

Inhibitory interneurons Fast-spiking interneurons were also modelled as multi-compartment Hodgkin-Huxley type neurons. The model was taken from Vierling-Claassen et al. [54] which can be found in ModelDB (*https://senselab.med.yale.edu/modeldb/ShowModel?model=141273*). The evolution of its membrane potential over time is governed by the following differential equation

$$C_m \frac{\partial V}{\partial t} = I_{\text{Nat}} + I_{\text{K}} + I_l + I_{axial},$$

where the currents are modelled with the same formalisms as for the pyramidal cells. The two ionic currents I_{Nat} and I_K were modelled as in [30].The basket cell model consisted of 16 compartments: the soma, and 15 dendritic compartments. The maximal conductance values $\bar{g}$ were different for different compartments and are subindexed as follows: s refers to the soma, d to the dendrite (for details see [54]). All values not shown are set to 0.

Fast inactivating Na$^+$ current, I_{Nat}

$$\alpha_m = -\frac{1}{\tau_{\text{ma}}} \cdot \frac{V_{\text{offm}} - V}{1 - \exp(\frac{(V_{\text{offm}} - V)}{V_{\text{slom}}})}$$

$$\beta_m = \frac{1}{\tau_{\text{mb}}} \cdot \frac{V_{\text{offm}} - V}{1 - \exp(\frac{-(V_{\text{offm}} - V)}{V_{\text{slom}}})}$$

$$\alpha_h = \frac{1}{\tau_{\text{ha}}} \cdot \frac{V_{\text{offh}} - V}{1 - \exp(\frac{(V_{\text{offh}} - V)}{V_{\text{sloh}}})}$$

$$\beta_h = -\frac{1}{\tau_{\text{hb}}} \cdot \frac{V_{\text{offh}} - V}{1 - \exp(\frac{-(V_{\text{offh}} - V)}{V_{\text{sloh}}})}$$

$$m_\infty = \frac{\alpha_m}{\alpha_m + \beta_m}$$

$$h_\infty = \frac{\alpha_h}{\alpha_h + \beta_h}$$

$$\tau_m = \frac{1}{T_{\text{adj}}(\alpha_m + \beta_m)}$$

$$\tau_h = \frac{1}{T_{\text{adj}}(\alpha_h + \beta_h)}$$

$V_{\text{offm}} = -25$ mV, $V_{\text{offh}} = -25$ mV, $V_{\text{slom}} = 9.0$ mV, $V_{\text{sloh}} = 9.0$ mV, $\tau_{\text{ma}} = 5.49$ ms, $\tau_{\text{mb}} = 8.06$ ms, $\tau_{\text{ha}} = 41.67$ ms, $\tau_{\text{hb}} = 109.89$ ms, $\bar{g}_s = 0.06$ S/cm^2, $\bar{g}_d = 0.035$ S/cm^2, $N_m = 3$, $N_h = 1$

K^+ current, I_{K}

$$\alpha_m = -\frac{1}{\tau_{\text{ma}}} \cdot \frac{V_{\text{offm}} - V}{1 - \exp(\frac{(V_{\text{offm}} - V)}{V_{\text{slom}}})}$$

$$\beta_m = \frac{1}{\tau_{\text{mb}}} \cdot \frac{V_{\text{offm}} - V}{1 - \exp(\frac{-(V_{\text{offm}} - V)}{V_{\text{slom}}})}$$

$$m_\infty = \frac{\alpha_m}{\alpha_m + \beta_m}$$

$$\tau_m = \frac{1}{T_{\text{adj}}(\alpha_m + \beta_m)}$$

$V_{\text{offm}} = -25$ mV, $V_{\text{offh}} = -25$ mV, $\tau_{\text{ma}} = 50.0$ ms, $\tau_{\text{mb}} = 500.0$ ms, $\bar{g}_s = 0.05$ S/cm^2, $\bar{g}_d = 0.035$ S/cm^2, $N_m = 1$.

Leak current, I_{leak}

$E = -73$ mV, $\bar{g}_s = 0.000012$ S/cm^2, $N_m = 0$, $N_h = 0$

For the interneurons, Na^+ and K^+ have a fixed reversal potential of $E_{Na} = 50\,\text{mV}$ and $E_{Na} = -85\,\text{mV}$.

1.1.2 Description of the Network Model

The network model consisted of 256 layer 5 pyramidal cells and 64 fast-spiking inhibitory interneurons and was implemented in NEURON [17] using the NetPyNE interface [12]. Pyramidal cells were connected to each other randomly with a probability of 0.06 (using AMPA and NMDA synaptic receptors). AMPA synaptic receptors were modelled as a double exponential function with a rise time of 0.1 ms and decay time of 3.0 ms. NMDA receptors were modelled as a first-order model including different binding and unbinding rates together with a magnesium block [10, 11]. AMPA receptors had a maximal conductance of 0.0012 nS and the NMDA receptors of 0.0006 nS, reflecting the higher contribution of AMPA receptors (see [43, 49]). Pyramidal cells were connected to inhibitory interneurons with a probability of 0.43, again using AMPA and NMDA receptors. However, for this connection type the maximal conductances for

AMPA receptors, 0.0012 nS, was substantially higher than for NMDA, 0.00013, reflecting the minor role NMDAergic activation plays in the recruitment of PV^+ interneurons [41, 49, 14]. Inhibitory interneurons formed connections with pyramidal cells and with themselves with a probability of 0.44 and 0.51, respectively. Inhibitory connections were realised using $GABA_A$ receptors, which were also modelled as double exponential functions with a rise time of 0.5 ms and a decay time of 8.0 ms (except for the condition of prolonged inhibitory decay times, where the decay time is increased to 25.0 ms). Maximal conductance values for inhibitory connections to pyramidal cells was 0.035 nS and to themselves 0.023 nS.

The network received two types of input: 1) Poissonian noise reflecting random cortical and subcortical background activity and 2) periodic input representing auditory steady-state stimuli at gamma frequency (operationalized at 40 Hz in this study). For the random background noise we utilized NEURON's built-in *NetStim* function with $rate = 200$, $noise = 1.0$ and $start = 500$. The parameters result in a noise stimulus reflecting a background rate of 200 Hz, that is completely random (i.e. Poissonian) which starts after 500 ms and lasts until the end of the simulation. The background *NetStim* element was connected to the pyramidal cells with a weight of 0.0325 and to the interneurons with a weight of 0.002. This setting resulted in asynchronous irregular firing of both cell populations at rates of 7.81 ± 0.07 Hz for the pyramidal cells and 6.18 ± 0.26 Hz for the inhibitory neurons, which is close to the reported spontaneous activity of $\sim$4.9 Hz reported for auditory cortex [20]. The periodic input resembling auditory click-train stimuli was also realised with NEURON's built-in *NetStim* function with $rate = 40.0$, $noise = 0.0$ and $start = 1000$. These parameters result in a

fully periodic stimulus with an inter-event interval of 25 ms, starting after 1000 ms and ending with the end of the simulation. In summary, the network was allowed to settle for the first 500 ms, then background input was switched on and after another 500 ms stimulus input was switched on. We simulated a total of 2000 ms and used only the last 1000 ms for the analysis of steady-state entrainment.

In our network model both, the background noise and the specific connections between neurons, are based on stochastic processes. To ensure that our findings do not depend on the specific instantiations of these stochastic processes, we always performed multiple simulations with different seeds for the random number generators underlying the processes. Specifically, we simulated 20 *subjects* by choosing 20 different seeds for the random number generator underlying the formation of synaptic connections, i.e. each *subject* had the same connectivity throughout all simulations. For each *subject* we then performed 10 *trials* by choosing 10 different random seeds for the random number generator underlying the background noise. In total, we performed 200 simulations for each network configuration, with the different network configurations being 1) the control network, 2) a single model variant, 3) a combination of different model variants, and 4) a synaptic alteration (either a reduced g_{max} or a increased τ_{decay} at GABAergic synapses).

1.1.3 Modelling SNP-like Genetic Variants

The single cell models include Hodgkin-Huxley type description for channel activation and inactivation, and hence, changes related to certain ion-channel-encoding gene variants that have been observed in experiments can be directly attributed to a change

of one or more parameters of these models. However, we only modelled changes to the pyramidal cells, which have been found to be strongly implicated by many schizophrenia-associated gene sets [48].

Here, we restricted ourselves to the following set of ion channel-encoding genes: CACNA1C, CACNA1D, CACNB2, CACNA1I, SCN1A and, HCN1. For details on the selected genes and variants see also [33, 34]. Supplementary Table 1 gives all details of the different variants of these genes and in Supplementary Tables 4 and 5 all details on how they were integrated into the pyramidal cell model are shown.

Extraction of Functional Genomics Studies The modelling of SNP-like genetic variants is based on an extensive search of the literature on how the genes CACNA1C, CACNA1D, CACNB2, CACNA1I, SCN1A, and HCN1 change the dynamics of the underlying ion channel. We included studies performed in different animal species and different cell types because of a current lack of data for a single animal and tissue type. Our inclusion criteria were as follows:

- The study applied a genetic variant of one of the genes of interest.
- The features of the variant-expressing cell were investigated using electrophysiology or Ca^{2+} imaging.
- The change from the control cell behaviour to the variant cell behaviour could be meaningfully translated to the layer 5 pyramidal cell model.
- The observed effect of the gene variant was not purely an effect of ion channel density or expression level.

We applied the last criterion mainly because there are numerous ways that might influence such an effect [46], while the changes of ion channel dynamics are supposedly more directly dependent on the genetic encoding. Supplementary table S1 lists all the pertinent data from the included studies [23, 9, 19, 50, 52, 51, 2, 59, 44, 45, 1, 27, 8, 36, 28, 21, 6, 53, 56, 5, 35, 22, 26, 57].

In studies that reported the effects of several variant types, the ranges of possible effects are considered. In case variants considered in such studies yielded positive and negative effects, both were included, however, if the reported endpoints of the ranges were too close to the control value (i.e. less than 1 mV or less than 10% of the distance between control value and the other endpoint), only the larger deviation was considered.

Scaling the Gene Variants Due to the polygenic nature of psychiatric disorders such as SCZ, it seems reasonable to assume that the disorder is not caused by a single SCZ-associated SNP but rather by a combination of sufficiently many (cf. [25]). Therefore, we followed earlier approaches [33, 34] to scale down the effect of single variants. We proceeded as follows. If a variant of the model (as shown in Supplementary Table 1) changed the response of the neuron too strongly, we scaled down the effect of the genetic variant until the differences between control and variant model neuron were within a given bound. Specifically, down-scaling was performed until the following five criteria were fulfilled ([33, 34]:

1. Exactly 4 spikes were induced as a response to current injection of 0.696 nA for the duration of 150 ms (square pulse),
2. exactly 1 spike was induced as a response to a distal (distance from soma = 620 μm) synaptic conductance (alpha-shaped with a time constant of 5 ms and a maximum amplitude of 0.0612 μS),
3. the neuron responded with exactly 2 spikes to a combined stimulus of a somatic current injection (1.137 nA, 10 ms, square pulse) and a distal synaptic conductance (alpha-shaped with a time constant of 5 ms and a maximum amplitude of 0.100 μS, applied 5 ms after the somatic pulse),
4. the integrated difference between the f-I curves of the variant model neuron and the control model neuron did not exceed 10% of the integral of the control neuron f-I curve, and

5. the membrane-potential limit cycle should not be too different from the control neuron limit cycle ($d_{cc}(lc1, lc2) \leq 600$, see [33] for the definition of the metric d_{cc}).

Here, the first three conditions constrain the magnitudes of transient responses of the neuron model and the amplitudes were chosen to guarantee the largest stability with respect to the response of the model using default parameters (stable here, means that an equal change in current amplitude on a logarithmic scale is needed to produce one action potential more or one action potential less.

In the case of a violation of one or more conditions, the effect of the variant on the parameters of the model was down-scaled to a fraction $c < 1$, where the violation is observed for the first time. The fraction for each considered variant can be found in Supplementary Table 4. If however an unscaled variant did not violate the conditions 1–5, we explored threshold effects up to twice the original effect, i.e., $c \leq 2$.

Since the parameters of the neuron model subject to change by the variants were very diverse, i.e. had various different roles and dimensions (mV, mM, ms, etc.), we adopted a careful scaling strategy. Parameters that could take positive and negative values were scaled linearly with the scaling factor c. Parameters that were exclusively non-negative, on the other hand, were scaled logarithmically with c. In detail, this meant that the difference in offset potentials (Voffm, Voffh) between the control model neuron and the variant model neuron, which are described by an additive term (i.e. $\pm \mathrm{x\,mV}$), was multiplied by the down-scaling parameter c. However, the change in all other parameters, which were described by multiplicative terms, was exponentiated

by the down-scaling factor c. This resulted in a continuous change of parameters for $c \in [0, 1]$, which is also directly extendable to down-scaling factors > 1.

Table 1: **Overview over the genetic variants.** The first column of the table shows the gene whose variant was studied in the named reference. Columns two and three show the current species and the affected model parameters, $offm$ and $offh$ representing the mid-points of activation and inactivation, respectively, $slom$ and $sloh$ their respective slopes, and $taum$ and $tauh$ their respective time constants. Multiple parameter changes in a single row are separated by a semicolon . The parameter names may refer to multiple model parameters: For example, for HVA Ca^{2+} currents, a change in $offm$ means a concurrent change in parameters V_{offma} and V_{offmb}, while $offh$ refers to parameters V_{offha} and V_{offhb}, $slom$ to V_{sloma} and V_{slomb}, $sloh$ to V_{sloha} and V_{slohb}, $taum$ to τ_{ma} and τ_{mb}, and $tauh$ to τ_{ha} and τ_{hb}. See model equations in Supplementary material for further details. The fourth column shows both direction and magnitude of the effect, where ±x mV refers to a change of the mid-point of the (in)activation curve by an absolute number, and ±x% refers to a percentual change in the underlying quantity. In many cases, studies considered more than one variant and here, they are categorized by the type of the variant where necessary (e.g., in [23] several variants of four loci, of which three were in pore-lining IS6 segment and two in bundle-crossing region of segment IIS6, were considered, and the variants are here categorized according to the segment they acted on). Column five gives the type of variant used, while the last column names the cell type under consideration.

Gene	Current	Parameter	Effect	Type of variant	Cell type
CACNA1C [23]	I_{CaHVA}	offm; offh	-25.9..-1.4mV; -27.0..-3.8mV	L429T, L434T, S435T, S435A, S435P	TSA201
CACNA1C [23]	I_{CaHVA}	offm; offh	-37.3..-9.7mV; -30.0..-11.8mV	L779T, I781T, I781P	TSA201
CACNA1C [9]	I_{CaHVA}	offm; offh	-31.4..+7.0mV; -28.5..+16.3mV	G432X, A780X, G1193X, A1503X	TSA201
		slom; sloh	-15..+45%; -28%..+38%		
CACNA1C [19]	I_{CaHVA}	offm	-38.5..+12.9mV	I781X, C769P, G770P, N771P, I773P	TSA201
		slom	-54..+56%	F778P, L779P, A780P, A782P, V783P	
CACNA1C [50]	I_{CaHVA}	offm; offh	-27.8..+8.7mV; -19.1..+4.7mV	I781T, N785A, N785G, N785L	TSA201
		slom	-11%..+14%		
CACNA1C [52]	I_{CaHVA}	offm; offh	-11.2..+1.0mV; -3.1..-0.3mV	Splice variants a1C77-A, -B, -C, and -D	TSA201
		sloh	+3%..+24%		

Table 2: **Overview over the genetic variants.** Table 1 continued

Gene		Current	Parameter	Effect	Type of variant	Cell type
CACNA1D	[51]	I_{CaHVA}	offm; offh	-10.9..-8.5mV; -3.0..+3.5mV	Splice variant 42A	TSA201 /
	[2]		slom; sloh	-27..-13%; -12..-19%		HEK293
			tauh	+25%		
CACNA1D	[51]	I_{CaHVA}	offm; offh	-10.6..+3.4mV; -5.3..+1.2mV	Splice variant 43S	TSA201 /
	[2]		slom; sloh	-20..+12%; -34..-8%		HEK293
			tauh	-28%		
CACNA1D	[59]	I_{CaHVA}	offm	+3.5..+6.6mV	Homozygous knockout	AV-node /
	[44]		slom; tauh	-25..+19%; -50..+12%		chromaffin
						cells
CACNA1D	[45]	I_{CaHVA}	offm; offh	-9.8mV; -15.4mV	A749G	TSA201
			slom; sloh	-20%; sloh		
CACNA1D	[1]	I_{CaHVA}	offm; offh	-24.2..+6.1mV; -14.5..-3.6mV	V259D, I750M, P1336R	TSA201
			slom; sloh	-30..+24%; -28%..+28%		
			tauh	+43%..+252%		
CACNA1D	[27]	I_{CaHVA}	offm	-17.8..-13.1mV	rCav1.3scg variant and mutants	TSA201
			slom; tauh	-19..-0%; -23%..+31%	7M2K, S244G, V1104A, and A2075V	
CACNB2	[8]	I_{CaHVA}	offh; sloh	-5.2mV; -31%	T11I	TSA201
CACNB2	[36]	I_{CaHVA}	taum	+70%	A1B2 vs A1 alone	HEK293
CACNB2	[28]	I_{CaHVA}	offm; offh	-4.9..+4.9mV; -5.1..+5.1mV	Splice variants N1, N3, N4, and N5	HEK293
			taum; tauh	-40%..+68%; -40%..+66%		
CACNB2	[21]	I_{CaHVA}	tauh	+26%	D601E	TSA201
CACNA1I	[42]	I_{CaLVA}	offm; offh	-0.2..+1.3mV; -0.5..+1.6mV	Alternative splicing of exons 9 and 33	HEK293
			taum; tauh	-13..+45%; -20..+8%		
CACNA1I	[13]	I_{CaLVA}	offm; offh	-4.3..-1.2mV; -4.4..-1.9mV	Truncated cDNAs L4, L6 and L9	HEK293
			slom; sloh	+5..+14%; -11%..+4%		
			taum; tauh	-47%..-15%; -54%..+1%		
CACNA1I	[13]	I_{CaLVA}	offm; offh	-4.3..-1.2mV; -4.4..-1.9mV	Truncated cDNAs L4, L6 and L9	HEK293
			slom; sloh	+5..+14%; -11%..+4%		
			taum; tauh	-47%..-15%; -54%..+1%		
SCN1A	[6]	I_{Nat}	offm; offh	-0.3mV; +5.0mV	Q1489K	Cultured
			slom; sloh	+15%; +23%		neocortical
					cells	
SCN1A	[53]	I_{Nat}	offm; offh	+2.8mV; +6.3..+9.6mV	L1649Q	TSA201
			slom; sloh	-1.6%; +4.2%		
SCN1A	[56]	I_{Nat}	offm; offh	-4.0mV; -5.8mV	R859H	TSA201
			slom; sloh; tauh	-8%; +13%; +43..+47%		
SCN1A	[56]	I_{Nat}	offm; offh	-8.1mV; +2.2mV	R865G	TSA201
			slom; sloh; tauh	-3%; -3%; +26..+59%		
SCN1A	[5]	I_{Nat}	offm; slom; tauh	+6.0mV; +16%; +29%	T1174S	TSA201
SCN1A	[35]	I_{Nat}	offm; offh	+10.0mV; -0.6mV	M145T	TSA201
			slom; sloh	+15%; +14%		
HCN1	[22]	I_h	offh; sloh	-2.1..-26.5mV; -12..-36%	D135W, D135H, D135N	HEK293
HCN1	[26]	I_h	offh	-25.9..+17.7mV	E229A, K230A, G231A, M232A, D233A	Oocytes
			sloh	-40..+3%	S234A, E235G, V236A, Y237A, EVY235-237DDD	
HCN1	[57]	I_h	offh; tauh	+2.4..+3.9mV; -12..-0%	WAG-HCN1, WAG-HCN1 + HCN1 co-expression	Oocytes

Table 3: **The effects of the genetic variants on model parameters.** The first column names the gene in which the gene variant was analyzed and the study where the effects were reported. The second column shows the effect of the variant on the model parameters, here $offm$ and $offh$ represent the midpoints of activation and inactivation, respectively, *slom* and *sloh* their respective slopes, and *taum* and *tauh* their respective time constants. The third column shows the down-scaling parameter resulting from the scaling procedure outlined above. The horizontal separation of rows refers to the different entries of Supplementary table 1: If the corresponding study showed a large range of effects on single model parameters, the endpoints of such ranges are here treated as separate variants and are therefore downscaled independently of each other. The variants shown in Figures 2, 3 and 4 are marked with an asterisk.

Gene	Parameter changes	Scaling parameter	
CACNA1C [23]	$V_{\mathrm{offm}*,\mathrm{CaHVA}}$: -25.9 mV; $V_{\mathrm{offh}*,\mathrm{CaHVA}}$: -27.0 mV	$c_{\mathrm{thresh}} = 0.066$	
CACNA1C [23]	$V_{\mathrm{offm}*,\mathrm{CaHVA}}$: -37.3 mV; $V_{\mathrm{offh}*,\mathrm{CaHVA}}$: -30.0 mV	$c_{\mathrm{thresh}} = 0.042$	
CACNA1C [9]	$V_{\mathrm{offm}*,\mathrm{CaHVA}}$: -31.4 mV; $V_{\mathrm{slom}*,\mathrm{CaHVA}}$: *0.85; $V_{\mathrm{offh}*,\mathrm{CaHVA}}$: -28.5 mV; $V_{\mathrm{sloh}*,\mathrm{CaHVA}}$: *0.72	$c_{\mathrm{thresh}} = 0.043$	
	$V_{\mathrm{offm}*,\mathrm{CaHVA}}$: +7.0 mV; $V_{\mathrm{slom}*,\mathrm{CaHVA}}$: *0.85; $V_{\mathrm{offh}*,\mathrm{CaHVA}}$: -28.5 mV; $V_{\mathrm{sloh}*,\mathrm{CaHVA}}$: *0.72	$c_{\mathrm{thresh}} = 0.101$	
	$V_{\mathrm{offm}*,\mathrm{CaHVA}}$: -31.4 mV; $V_{\mathrm{slom}*,\mathrm{CaHVA}}$: *1.45; $V_{\mathrm{offh}*,\mathrm{CaHVA}}$: -28.5 mV; $V_{\mathrm{sloh}*,\mathrm{CaHVA}}$: *0.72	$c_{\mathrm{thresh}} = 0.049$	
	$V_{\mathrm{offm}*,\mathrm{CaHVA}}$: +7.0 mV; $V_{\mathrm{slom}*,\mathrm{CaHVA}}$: *1.45; $V_{\mathrm{offh}*,\mathrm{CaHVA}}$: -28.5 mV; $V_{\mathrm{sloh}*,\mathrm{CaHVA}}$: *0.72	$c_{\mathrm{thresh}} = 0.76$	
	$V_{\mathrm{offm}*,\mathrm{CaHVA}}$: -31.4 mV; $V_{\mathrm{slom}*,\mathrm{CaHVA}}$: *0.85; $V_{\mathrm{offh}*,\mathrm{CaHVA}}$: +16.3 mV; $V_{\mathrm{sloh}*,\mathrm{CaHVA}}$: *0.72	$c_{\mathrm{thresh}} = 0.076$	*
	$V_{\mathrm{offm}*,\mathrm{CaHVA}}$: +7.0 mV; $V_{\mathrm{slom}*,\mathrm{CaHVA}}$: *0.85; $V_{\mathrm{offh}*,\mathrm{CaHVA}}$: +16.3 mV; $V_{\mathrm{sloh}*,\mathrm{CaHVA}}$: *0.72	$c_{\mathrm{thresh}} = 0.693$	
	$V_{\mathrm{offm}*,\mathrm{CaHVA}}$: -31.4 mV; $V_{\mathrm{slom}*,\mathrm{CaHVA}}$: *1.45; $V_{\mathrm{offh}*,\mathrm{CaHVA}}$: +16.3 mV; $V_{\mathrm{sloh}*,\mathrm{CaHVA}}$: *0.72	$c_{\mathrm{thresh}} = 0.034$	
	$V_{\mathrm{offm}*,\mathrm{CaHVA}}$: +7.0 mV; $V_{\mathrm{slom}*,\mathrm{CaHVA}}$: *1.45; $V_{\mathrm{offh}*,\mathrm{CaHVA}}$: +16.3 mV; $V_{\mathrm{sloh}*,\mathrm{CaHVA}}$: *0.72	$c_{\mathrm{thresh}} = 0.359$	
	$V_{\mathrm{offm}*,\mathrm{CaHVA}}$: +7.0 mV; $V_{\mathrm{slom}*,\mathrm{CaHVA}}$: *0.85; $V_{\mathrm{offh}*,\mathrm{CaHVA}}$: -28.5 mV; $V_{\mathrm{sloh}*,\mathrm{CaHVA}}$: *1.38	$c_{\mathrm{thresh}} = 0.059$	
	$V_{\mathrm{offm}*,\mathrm{CaHVA}}$: -31.4 mV; $V_{\mathrm{slom}*,\mathrm{CaHVA}}$: *1.45; $V_{\mathrm{offh}*,\mathrm{CaHVA}}$: -28.5 mV; $V_{\mathrm{sloh}*,\mathrm{CaHVA}}$: *1.38	$c_{\mathrm{thresh}} = 0.103$	
	$V_{\mathrm{offm}*,\mathrm{CaHVA}}$: +7.0 mV; $V_{\mathrm{slom}*,\mathrm{CaHVA}}$: *1.45; $V_{\mathrm{offh}*,\mathrm{CaHVA}}$: -28.5 mV; $V_{\mathrm{sloh}*,\mathrm{CaHVA}}$: *1.38	$c_{\mathrm{thresh}} = 0.049$	
	$V_{\mathrm{offm}*,\mathrm{CaHVA}}$: +7.0 mV; $V_{\mathrm{slom}*,\mathrm{CaHVA}}$: *0.85; $V_{\mathrm{offh}*,\mathrm{CaHVA}}$: +16.3 mV; $V_{\mathrm{sloh}*,\mathrm{CaHVA}}$: *1.38	$c_{\mathrm{thresh}} = 0.176$	
	$V_{\mathrm{offm}*,\mathrm{CaHVA}}$: -31.4 mV; $V_{\mathrm{slom}*,\mathrm{CaHVA}}$: *1.45; $V_{\mathrm{offh}*,\mathrm{CaHVA}}$: +16.3 mV; $V_{\mathrm{sloh}*,\mathrm{CaHVA}}$: *1.38	$c_{\mathrm{thresh}} = 0.038$	
	$V_{\mathrm{offm}*,\mathrm{CaHVA}}$: +7.0 mV; $V_{\mathrm{slom}*,\mathrm{CaHVA}}$: *1.45; $V_{\mathrm{offh}*,\mathrm{CaHVA}}$: +16.3 mV; $V_{\mathrm{sloh}*,\mathrm{CaHVA}}$: *1.38	$c_{\mathrm{thresh}} = 0.113$	
CACNA1C [19]	$V_{\mathrm{offm}*,\mathrm{CaHVA}}$: -38.5 mV; $V_{\mathrm{slom}*,\mathrm{CaHVA}}$: *0.46	$c_{\mathrm{thresh}} = 0.028$	
	$V_{\mathrm{offm}*,\mathrm{CaHVA}}$: +12.9 mV; $V_{\mathrm{slom}*,\mathrm{CaHVA}}$: *0.46	$c_{\mathrm{thresh}} = 0.123$	
CACNA1C [50]	$V_{\mathrm{offm}*,\mathrm{CaHVA}}$: -27.8 mV; $V_{\mathrm{slom}*,\mathrm{CaHVA}}$: *0.89; $V_{\mathrm{offh}*,\mathrm{CaHVA}}$: -19.1 mV	$c_{\mathrm{thresh}} = 0.052$	
	$V_{\mathrm{offm}*,\mathrm{CaHVA}}$: +8.7 mV; $V_{\mathrm{slom}*,\mathrm{CaHVA}}$: *0.89; $V_{\mathrm{offh}*,\mathrm{CaHVA}}$: -19.1 mV	$c_{\mathrm{thresh}} = 0.077$	
	$V_{\mathrm{offm}*,\mathrm{CaHVA}}$: -27.8 mV; $V_{\mathrm{slom}*,\mathrm{CaHVA}}$: *1.14; $V_{\mathrm{offh}*,\mathrm{CaHVA}}$: -19.1 mV	$c_{\mathrm{thresh}} = 0.057$	
	$V_{\mathrm{offm}*,\mathrm{CaHVA}}$: +8.7 mV; $V_{\mathrm{slom}*,\mathrm{CaHVA}}$: *1.14; $V_{\mathrm{offh}*,\mathrm{CaHVA}}$: -19.1 mV	$c_{\mathrm{thresh}} = 0.069$	
	$V_{\mathrm{offm}*,\mathrm{CaHVA}}$: -27.8 mV; $V_{\mathrm{slom}*,\mathrm{CaHVA}}$: *0.89; $V_{\mathrm{offh}*,\mathrm{CaHVA}}$: +4.7 mV	$c_{\mathrm{thresh}} = 0.042$	
	$V_{\mathrm{offm}*,\mathrm{CaHVA}}$: +8.7 mV; $V_{\mathrm{slom}*,\mathrm{CaHVA}}$: *0.89; $V_{\mathrm{offh}*,\mathrm{CaHVA}}$: +4.7 mV	$c_{\mathrm{thresh}} = 0.145$	
	$V_{\mathrm{offm}*,\mathrm{CaHVA}}$: -27.8 mV; $V_{\mathrm{slom}*,\mathrm{CaHVA}}$: *1.14; $V_{\mathrm{offh}*,\mathrm{CaHVA}}$: +4.7 mV	$c_{\mathrm{thresh}} = 0.044$	
	$V_{\mathrm{offm}*,\mathrm{CaHVA}}$: +8.7 mV; $V_{\mathrm{slom}*,\mathrm{CaHVA}}$: *1.14; $V_{\mathrm{offh}*,\mathrm{CaHVA}}$: +4.7 mV	$c_{\mathrm{thresh}} = 0.119$	

Table 4: Table 3 continued.

Gene	Parameter changes	Scaling parameter
CACNA1C [52]	$V_{\mathrm{offm}*,\mathrm{CaHVA}}$: -11.2 mV; $V_{\mathrm{offh}*,\mathrm{CaHVA}}$: -3.1 mV; $V_{\mathrm{sloh}*,\mathrm{CaHVA}}$: *1.24	$c_{\mathrm{thresh}} = 0.157$
	$V_{\mathrm{offm}*,\mathrm{CaHVA}}$: +1.0 mV; $V_{\mathrm{offh}*,\mathrm{CaHVA}}$: -3.1 mV; $V_{\mathrm{sloh}*,\mathrm{CaHVA}}$: *1.24	$c_{\mathrm{thresh}} = 0.236$
CACNA1D [51],	$V_{\mathrm{offm}*,\mathrm{CaHVA}}$: -10.9 mV; $V_{\mathrm{slom}*,\mathrm{CaHVA}}$: *0.73; $V_{\mathrm{offh}*,\mathrm{CaHVA}}$: -3.0 mV; $V_{\mathrm{sloh}*,\mathrm{CaHVA}}$: *0.81; $\tau_{\mathrm{h}*,\mathrm{CaHVA}}$: *1.25	$c_{\mathrm{thresh}} = 0.083$
[2]	$V_{\mathrm{offm}*,\mathrm{CaHVA}}$: -10.9 mV; $V_{\mathrm{slom}*,\mathrm{CaHVA}}$: *0.73; $V_{\mathrm{offh}*,\mathrm{CaHVA}}$: +3.5 mV; $V_{\mathrm{sloh}*,\mathrm{CaHVA}}$: *0.81; $\tau_{\mathrm{h}*,\mathrm{CaHVA}}$: *1.25	$c_{\mathrm{thresh}} = 0.075$
CACNA1D [51],	$V_{\mathrm{offm}*,\mathrm{CaHVA}}$: -10.6 mV; $V_{\mathrm{slom}*,\mathrm{CaHVA}}$: *0.8; $V_{\mathrm{offh}*,\mathrm{CaHVA}}$: -5.3 mV; $V_{\mathrm{sloh}*,\mathrm{CaHVA}}$: *0.66; $\tau_{\mathrm{h}*,\mathrm{CaHVA}}$: *0.72	$c_{\mathrm{thresh}} = 0.080$
[2]	$V_{\mathrm{offm}*,\mathrm{CaHVA}}$: +3.4 mV; $V_{\mathrm{slom}*,\mathrm{CaHVA}}$: *0.8; $V_{\mathrm{offh}*,\mathrm{CaHVA}}$: -5.3 mV; $V_{\mathrm{sloh}*,\mathrm{CaHVA}}$: *0.66; $\tau_{\mathrm{h}*,\mathrm{CaHVA}}$: *0.72	$c_{\mathrm{thresh}} = 1.962$
	$V_{\mathrm{offm}*,\mathrm{CaHVA}}$: -10.6 mV; $V_{\mathrm{slom}*,\mathrm{CaHVA}}$: *1.12; $V_{\mathrm{offh}*,\mathrm{CaHVA}}$: -5.3 mV; $V_{\mathrm{sloh}*,\mathrm{CaHVA}}$: *0.66; $\tau_{\mathrm{h}*,\mathrm{CaHVA}}$: *0.72	$c_{\mathrm{thresh}} = 0.094$
	$V_{\mathrm{offm}*,\mathrm{CaHVA}}$: +3.4 mV; $V_{\mathrm{slom}*,\mathrm{CaHVA}}$: *1.12; $V_{\mathrm{offh}*,\mathrm{CaHVA}}$: -5.3 mV; $V_{\mathrm{sloh}*,\mathrm{CaHVA}}$: *0.66; $\tau_{\mathrm{h}*,\mathrm{CaHVA}}$: *0.72	$c_{\mathrm{thresh}} = 0.905$
	$V_{\mathrm{offm}*,\mathrm{CaHVA}}$: -10.6 mV; $V_{\mathrm{slom}*,\mathrm{CaHVA}}$: *0.8; $V_{\mathrm{offh}*,\mathrm{CaHVA}}$: +1.2 mV; $V_{\mathrm{sloh}*,\mathrm{CaHVA}}$: *0.66; $\tau_{\mathrm{h}*,\mathrm{CaHVA}}$: *0.72	$c_{\mathrm{thresh}} = 0.072$
	$V_{\mathrm{offm}*,\mathrm{CaHVA}}$: +3.4 mV; $V_{\mathrm{slom}*,\mathrm{CaHVA}}$: *0.8; $V_{\mathrm{offh}*,\mathrm{CaHVA}}$: +1.2 mV; $V_{\mathrm{sloh}*,\mathrm{CaHVA}}$: *0.66; $\tau_{\mathrm{h}*,\mathrm{CaHVA}}$: *0.72	$c_{\mathrm{thresh}} = 0.386$
	$V_{\mathrm{offm}*,\mathrm{CaHVA}}$: -10.6 mV; $V_{\mathrm{slom}*,\mathrm{CaHVA}}$: *1.12; $V_{\mathrm{offh}*,\mathrm{CaHVA}}$: +1.2 mV; $V_{\mathrm{sloh}*,\mathrm{CaHVA}}$: *0.66; $\tau_{\mathrm{h}*,\mathrm{CaHVA}}$: *0.72	$c_{\mathrm{thresh}} = 0.083$
	$V_{\mathrm{offm}*,\mathrm{CaHVA}}$: +3.4 mV; $V_{\mathrm{slom}*,\mathrm{CaHVA}}$: *1.12; $V_{\mathrm{offh}*,\mathrm{CaHVA}}$: +1.2 mV; $V_{\mathrm{sloh}*,\mathrm{CaHVA}}$: *0.66; $\tau_{\mathrm{h}*,\mathrm{CaHVA}}$: *0.72	$c_{\mathrm{thresh}} = 1.117$
CACNA1D [59],	$V_{\mathrm{offm}*,\mathrm{CaHVA}}$: +6.6 mV; $V_{\mathrm{slom}*,\mathrm{CaHVA}}$: *0.75; $\tau_{\mathrm{h}*,\mathrm{CaHVA}}$: *0.5	$c_{\mathrm{thresh}} = 0.190$
[44]	$V_{\mathrm{offm}*,\mathrm{CaHVA}}$: +6.6 mV; $V_{\mathrm{slom}*,\mathrm{CaHVA}}$: *1.19; $\tau_{\mathrm{h}*,\mathrm{CaHVA}}$: *0.5	$c_{\mathrm{thresh}} = 0.123$
	$V_{\mathrm{offm}*,\mathrm{CaHVA}}$: +6.6 mV; $V_{\mathrm{slom}*,\mathrm{CaHVA}}$: *0.75; $\tau_{\mathrm{h}*,\mathrm{CaHVA}}$: *1.12	$c_{\mathrm{thresh}} = 0.209$
	$V_{\mathrm{offm}*,\mathrm{CaHVA}}$: +6.6 mV; $V_{\mathrm{slom}*,\mathrm{CaHVA}}$: *1.19; $\tau_{\mathrm{h}*,\mathrm{CaHVA}}$: *1.12	$c_{\mathrm{thresh}} = 0.130$
CACNA1D [45]	$V_{\mathrm{offm}*,\mathrm{CaHVA}}$: -9.8 mV; $V_{\mathrm{slom}*,\mathrm{CaHVA}}$: *0.8; $V_{\mathrm{offh}*,\mathrm{CaHVA}}$: -15.4 mV; $V_{\mathrm{sloh}*,\mathrm{CaHVA}}$: *1.05	$c_{\mathrm{thresh}} = 0.181$
CACNA1D [1]	$V_{\mathrm{offm}*,\mathrm{CaHVA}}$: -24.2 mV; $V_{\mathrm{slom}*,\mathrm{CaHVA}}$: *0.7; $V_{\mathrm{offh}*,\mathrm{CaHVA}}$: -14.5 mV; $V_{\mathrm{sloh}*,\mathrm{CaHVA}}$: *0.72; $\tau_{\mathrm{h}*,\mathrm{CaHVA}}$: *3.52	$c_{\mathrm{thresh}} = 0.045$
	$V_{\mathrm{offm}*,\mathrm{CaHVA}}$: +6.1 mV; $V_{\mathrm{slom}*,\mathrm{CaHVA}}$: *0.7; $V_{\mathrm{offh}*,\mathrm{CaHVA}}$: -14.5 mV; $V_{\mathrm{sloh}*,\mathrm{CaHVA}}$: *0.72; $\tau_{\mathrm{h}*,\mathrm{CaHVA}}$: *3.52	$c_{\mathrm{thresh}} = 0.318$
	$V_{\mathrm{offm}*,\mathrm{CaHVA}}$: -24.2 mV; $V_{\mathrm{slom}*,\mathrm{CaHVA}}$: *1.24; $V_{\mathrm{offh}*,\mathrm{CaHVA}}$: -14.5 mV; $V_{\mathrm{sloh}*,\mathrm{CaHVA}}$: *0.72; $\tau_{\mathrm{h}*,\mathrm{CaHVA}}$: *3.52	$c_{\mathrm{thresh}} = 0.053$
	$V_{\mathrm{offm}*,\mathrm{CaHVA}}$: +6.1 mV; $V_{\mathrm{slom}*,\mathrm{CaHVA}}$: *1.24; $V_{\mathrm{offh}*,\mathrm{CaHVA}}$: -14.5 mV; $V_{\mathrm{sloh}*,\mathrm{CaHVA}}$: *0.72; $\tau_{\mathrm{h}*,\mathrm{CaHVA}}$: *3.52	$c_{\mathrm{thresh}} = 0.152$
	$V_{\mathrm{offm}*,\mathrm{CaHVA}}$: -24.2 mV; $V_{\mathrm{slom}*,\mathrm{CaHVA}}$: *0.7; $V_{\mathrm{offh}*,\mathrm{CaHVA}}$: -14.5 mV; $V_{\mathrm{sloh}*,\mathrm{CaHVA}}$: *1.28; $\tau_{\mathrm{h}*,\mathrm{CaHVA}}$: *3.52	$c_{\mathrm{thresh}} = 0.059$
	$V_{\mathrm{offm}*,\mathrm{CaHVA}}$: +6.1 mV; $V_{\mathrm{slom}*,\mathrm{CaHVA}}$: *0.7; $V_{\mathrm{offh}*,\mathrm{CaHVA}}$: -14.5 mV; $V_{\mathrm{sloh}*,\mathrm{CaHVA}}$: *1.28; $\tau_{\mathrm{h}*,\mathrm{CaHVA}}$: *3.52	$c_{\mathrm{thresh}} = 0.105$
	$V_{\mathrm{offm}*,\mathrm{CaHVA}}$: -24.2 mV; $V_{\mathrm{slom}*,\mathrm{CaHVA}}$: *1.24; $V_{\mathrm{offh}*,\mathrm{CaHVA}}$: -14.5 mV; $V_{\mathrm{sloh}*,\mathrm{CaHVA}}$: *1.28; $\tau_{\mathrm{h}*,\mathrm{CaHVA}}$: *3.52	$c_{\mathrm{thresh}} = 0.074$
	$V_{\mathrm{offm}*,\mathrm{CaHVA}}$: +6.1 mV; $V_{\mathrm{slom}*,\mathrm{CaHVA}}$: *1.24; $V_{\mathrm{offh}*,\mathrm{CaHVA}}$: -14.5 mV; $V_{\mathrm{sloh}*,\mathrm{CaHVA}}$: *1.28; $\tau_{\mathrm{h}*,\mathrm{CaHVA}}$: *3.52	$c_{\mathrm{thresh}} = 0.076$

Table 5: Table 4 continued.

Gene	Parameter changes	Scaling parameter	
CACNA1D [27]	$V_{\mathrm{offm*,CaHVA}}$: -17.8 mV; $V_{\mathrm{slom*,CaHVA}}$: *0.81; $\tau_{\mathrm{h*,CaHVA}}$: *0.77	c_{thresh} = 0.065	
	$V_{\mathrm{offm*,CaHVA}}$: -17.8 mV; $V_{\mathrm{slom*,CaHVA}}$: *0.81; $\tau_{\mathrm{h*,CaHVA}}$: *1.31	c_{thresh} = 0.063	
CACNB2 [8]	$V_{\mathrm{offh*,CaHVA}}$: -5.2 mV; $V_{\mathrm{sloh*,CaHVA}}$: *0.69	c_{thresh} = 0.381	
CACNB2 [36]	$\tau_{\mathrm{h*,CaHVA}}$: *1.7	c_{thresh} = 2.000	
CACNB2 [28]	$V_{\mathrm{offm*,CaHVA}}$: -4.9 mV; $V_{\mathrm{offh*,CaHVA}}$: -5.1 mV; $\tau_{\mathrm{m*,CaHVA}}$: *0.6; $\tau_{\mathrm{h*,CaHVA}}$: *0.6	c_{thresh} = 0.194	
	$V_{\mathrm{offm*,CaHVA}}$: +4.9 mV; $V_{\mathrm{offh*,CaHVA}}$: -5.1 mV; $\tau_{\mathrm{m*,CaHVA}}$: *0.6; $\tau_{\mathrm{h*,CaHVA}}$: *0.6	c_{thresh} = 0.157	
	$V_{\mathrm{offm*,CaHVA}}$: -4.9 mV; $V_{\mathrm{offh*,CaHVA}}$: +5.1 mV; $\tau_{\mathrm{m*,CaHVA}}$: *0.6; $\tau_{\mathrm{h*,CaHVA}}$: *0.6	c_{thresh} = 0.122	
	$V_{\mathrm{offm*,CaHVA}}$: +4.9 mV; $V_{\mathrm{offh*,CaHVA}}$: +5.1 mV; $\tau_{\mathrm{m*,CaHVA}}$: *0.6; $\tau_{\mathrm{h*,CaHVA}}$: *0.6	c_{thresh} = 1.101	
	$V_{\mathrm{offm*,CaHVA}}$: -4.9 mV; $V_{\mathrm{offh*,CaHVA}}$: -5.1 mV; $\tau_{\mathrm{m*,CaHVA}}$: *1.68; $\tau_{\mathrm{h*,CaHVA}}$: *0.6	c_{thresh} = 0.814	
	$V_{\mathrm{offm*,CaHVA}}$: +4.9 mV; $V_{\mathrm{offh*,CaHVA}}$: -5.1 mV; $\tau_{\mathrm{m*,CaHVA}}$: *1.68; $\tau_{\mathrm{h*,CaHVA}}$: *0.6	c_{thresh} = 0.146	
	$V_{\mathrm{offm*,CaHVA}}$: -4.9 mV; $V_{\mathrm{offh*,CaHVA}}$: +5.1 mV; $\tau_{\mathrm{m*,CaHVA}}$: *1.68; $\tau_{\mathrm{h*,CaHVA}}$: *0.6	c_{thresh} = 0.517	
	$V_{\mathrm{offm*,CaHVA}}$: +4.9 mV; $V_{\mathrm{offh*,CaHVA}}$: +5.1 mV; $\tau_{\mathrm{m*,CaHVA}}$: *1.68; $\tau_{\mathrm{h*,CaHVA}}$: *0.6	c_{thresh} = 0.204	
	$V_{\mathrm{offm*,CaHVA}}$: -4.9 mV; $V_{\mathrm{offh*,CaHVA}}$: -5.1 mV; $\tau_{\mathrm{m*,CaHVA}}$: *0.6; $\tau_{\mathrm{h*,CaHVA}}$: *1.66	c_{thresh} = 0.285	
	$V_{\mathrm{offm*,CaHVA}}$: +4.9 mV; $V_{\mathrm{offh*,CaHVA}}$: -5.1 mV; $\tau_{\mathrm{m*,CaHVA}}$: *0.6; $\tau_{\mathrm{h*,CaHVA}}$: *1.66	c_{thresh} = 0.511	
	$V_{\mathrm{offm*,CaHVA}}$: -4.9 mV; $V_{\mathrm{offh*,CaHVA}}$: +5.1 mV; $\tau_{\mathrm{m*,CaHVA}}$: *0.6; $\tau_{\mathrm{h*,CaHVA}}$: *1.66	c_{thresh} = 0.197	
	$V_{\mathrm{offm*,CaHVA}}$: +4.9 mV; $V_{\mathrm{offh*,CaHVA}}$: +5.1 mV; $\tau_{\mathrm{m*,CaHVA}}$: *0.6; $\tau_{\mathrm{h*,CaHVA}}$: *1.66	c_{thresh} = 1.687	
	$V_{\mathrm{offm*,CaHVA}}$: -4.9 mV; $V_{\mathrm{offh*,CaHVA}}$: -5.1 mV; $\tau_{\mathrm{m*,CaHVA}}$: *1.68; $\tau_{\mathrm{h*,CaHVA}}$: *1.66	c_{thresh} = 0.707	
	$V_{\mathrm{offm*,CaHVA}}$: +4.9 mV; $V_{\mathrm{offh*,CaHVA}}$: -5.1 mV; $\tau_{\mathrm{m*,CaHVA}}$: *1.68; $\tau_{\mathrm{h*,CaHVA}}$: *1.66	c_{thresh} = 0.156	
	$V_{\mathrm{offm*,CaHVA}}$: -4.9 mV; $V_{\mathrm{offh*,CaHVA}}$: +5.1 mV; $\tau_{\mathrm{m*,CaHVA}}$: *1.68; $\tau_{\mathrm{h*,CaHVA}}$: *1.66	c_{thresh} = 0.460	
	$V_{\mathrm{offm*,CaHVA}}$: +4.9 mV; $V_{\mathrm{offh*,CaHVA}}$: +5.1 mV; $\tau_{\mathrm{m*,CaHVA}}$: *1.68; $\tau_{\mathrm{h*,CaHVA}}$: *1.66	c_{thresh} = 0.218	
CACNB2 [21]	$\tau_{\mathrm{h*,CaHVA}}$: *1.26	c_{thresh} = 2.000	
CACNA1I [42]	$V_{\mathrm{offma,CaLVA}}$: +1.3 mV; $V_{\mathrm{offha,CaLVA}}$: +1.6 mV; $\tau_{\mathrm{m*,CaLVA}}$: *0.87; $\tau_{\mathrm{h*,CaLVA}}$: *0.8	c_{thresh} = 2.000	
	$V_{\mathrm{offma,CaLVA}}$: +1.3 mV; $V_{\mathrm{offha,CaLVA}}$: +1.6 mV; $\tau_{\mathrm{m*,CaLVA}}$: *1.45; $\tau_{\mathrm{h*,CaLVA}}$: *0.8	c_{thresh} = 2.000	*
CACNA1I [13]	$V_{\mathrm{offma,CaLVA}}$: -4.3 mV; $V_{\mathrm{sloma,CaLVA}}$: *1.14; $V_{\mathrm{offha,CaLVA}}$: -4.4 mV; $V_{\mathrm{sloha,CaLVA}}$: *0.89; $\tau_{\mathrm{m*,CaLVA}}$: *0.53; $\tau_{\mathrm{h*,CaLVA}}$: *0.46	c_{thresh} = 0.968	
	$V_{\mathrm{offma,CaLVA}}$: -4.3 mV; $V_{\mathrm{sloma,CaLVA}}$: *1.14; $V_{\mathrm{offha,CaLVA}}$: -4.4 mV; $V_{\mathrm{sloha,CaLVA}}$: *1.04; $\tau_{\mathrm{m*,CaLVA}}$: *0.53; $\tau_{\mathrm{h*,CaLVA}}$: *0.46	c_{thresh} = 2.000	
SCN1A [6]	$V_{\mathrm{offm,Nat}}$: -0.3 mV; $V_{\mathrm{offh,Nat}}$: +5 mV; $V_{\mathrm{slom,Nat}}$: *1.15; $V_{\mathrm{sloh,Nat}}$: *1.23	c_{thresh} = 0.049	
SCN1A [53]	$V_{\mathrm{offm,Nat}}$: +2.8 mV; $V_{\mathrm{offh,Nat}}$: +9.6 mV; $V_{\mathrm{slom,Nat}}$: *0.984; $V_{\mathrm{sloh,Nat}}$: *1.042	c_{thresh} = 0.063	
SCN1A [56]	$V_{\mathrm{offm,Nat}}$: -4.0 mV; $V_{\mathrm{offh,Nat}}$: -5.8 mV; $V_{\mathrm{slom,Nat}}$: *0.92; $V_{\mathrm{sloh,Nat}}$: *1.13; $\tau_{\mathrm{h*,Nat}}$: *1.47	c_{thresh} = 0.273	
SCN1A [56]	$V_{\mathrm{offm,Nat}}$: -8.1 mV; $V_{\mathrm{offh,Nat}}$: +2.2 mV; $V_{\mathrm{slom,Nat}}$: *0.97; $V_{\mathrm{sloh,Nat}}$: *0.97; $\tau_{\mathrm{h*,Nat}}$: *1.59	c_{thresh} = 0.037	
SCN1A [5]	$V_{\mathrm{offm,Nat}}$: +6.0 mV; $V_{\mathrm{slom,Nat}}$: *1.16; $\tau_{\mathrm{h*,Nat}}$: *1.29	c_{thresh} = 0.129	
SCN1A [35]	$V_{\mathrm{offm,Nat}}$: +10.0 mV; $V_{\mathrm{offh,Nat}}$: -0.6 mV; $V_{\mathrm{slom,Nat}}$: *1.15; $V_{\mathrm{sloh,Nat}}$: *1.14	c_{thresh} = 0.062	
HCN1 [22]	$V_{\mathrm{offm*,h}}$: -26.5 mV; $V_{\mathrm{slom*,h}}$: *0.64	c_{thresh} = 0.296	*
HCN1 [26]	$V_{\mathrm{offm*,h}}$: -25.9 mV; $V_{\mathrm{slom*,h}}$: *0.6	c_{thresh} = 0.282	*
	$V_{\mathrm{offm*,h}}$: +17.7 mV; $V_{\mathrm{slom*,h}}$: *0.6	c_{thresh} = 0.807	
HCN1 [57]	$V_{\mathrm{offm*,h}}$: +3.9 mV; $\tau_{\mathrm{m*,h}}$: *0.88	c_{thresh} = 1.226	

1.1.4 Synaptic Alterations

ASSR deficits in schizophrenia have previously been attributed to changes in synaptic transmission, mainly at GABAergic synapses [15]. To compare the effects of the cell-intrinsic changes to excitability introduced by the genetic variants considered in this study to these alterations at the synaptic level, we implemented the two most commonly modelled synaptic alterations [55, 39]: 1) a reduction of GABA levels due to a decrease in the expression of GAD67 [15], a GABA precursor, operationalized as a 25% reduction of the maximal GABA conductance g_{max} at GABAergic synapses (referred to as the *Gmax* condition) and 2) an increase of the decay time constant τ_{decay} at GABAergic synapses, as a result of a decrease of GAT1 [15], an enzyme responsible for GABA reuptake, operationalized as an increase from 8 ms to 25 ms (referred to as the *IPSC* condition).

1.1.5 Data analysis

The simulated local field potential (LFP) was recorded using NetPyNE's LFP recording capabilities with a recording time step of 0.1 ms. From the simulated LFP signals we calculated two measures capturing the degree of synchronous network activity: 1) the power spectral density and 2) the inter-trial phase coherence of the entrained network oscillation.

To compute the power spectral density, we used Welch's periodogram method (using the implementation in matplotlib.pyplot). Specifically, the power spectral density P_{xx}

of the signal is computed as

$$P_{xx} = |fft(x)|^2$$

where x is the input signal and fft the Fast-Fourier Transform.

For the inter-trial phase coherence, we band-pass filtered the simulated LFP signal around the frequency of interest, i.e. 40 Hz ±2.5 Hz, and computed an analytic signal using the Hilbert transform. The analytic signal $x_a(t)$ can be expressed in polar coordinates as

$$x_a(t) = x_m(t)e^{j\phi(t)}$$

where $x_m(t)$ is called the instantaneous amplitude or envelope and $\phi(t)$ is called the instantaneous phase. We calculated the instantaneous phase $\phi_k(t)$ for each trial k of each subject j and then, for each subject j, the inter-trial phase coherence was calculated as

$$ITC(j,t) = |\frac{1}{N}\sum_{k=1}^{N} e^{i\phi_k(t)}|$$

We then averaged the individual inter-trial phase coherences over time. The ITC is a normalized measure for which a value of 0 reflects maximal variability of phases across trials and a value of 1 no variability.

To further understand the effect the changes to individual parameters have on the gamma power, we correlated (Pearson correlation) the parameter changes (i.e. changes to $offm$, $offh$, $slom$, $sloh$, $taum$ and $tauh$), for the model variants affecting the HVA Ca^{2+} channel with the change in evoked gamma power. Since we only included relatively few model variants affecting the other channels (LVA Ca^{2+}, Nat and h), we omitted such an analysis for those channels.

Comparison of single model variants, combinations of model variants and synaptic alterations against the shared control was performed using Cumming estimation plots (see Figures 2, 3, and 4) and permutation t-tests (see Supplementary Table 6) using the DABEST Python package [18]. 5000 bootstrap samples were taken; the confidence interval was bias-corrected and accelerated. The p values reported are the likelihoods of observing the mean differences, if the null hypothesis of zero difference is true. For each permutation p value, 5000 reshuffles of the control and test labels were performed. For more details see [18].

To compare our findings with our earlier findings [34], we calculated Pearson correlation coefficients between gamma power and resonance power in the delta band and PPI threshold, respectively. Specifically, resonance power in the delta band from our earlier study was calculated as the median of the power spectrum amplitude at a baseline frequency of 1.5 Hz. The PPI threshold is the threshold synaptic conductance of 3000 simultaneously activated synapses for generating a second action potential if a suprathreshold stimulus was given to the same synapses 60 ms before. For further details see [34]. We have to note that resonance power in the delta band in our earlier work was generated in a substantially different network model, although based on the same single cell model of layer 5 pyramidal cells, and that the PPI thresholds were generated with the full layer 5 pyramidal cell model from Hay et al. [16]. Importantly, the modelling of the SNP-like genetic variants followed exactly the same procedure as outlined here.

1.2 Supplementary Results

1.2.1 Single Cell Behaviour

The response of the two single cell models in response to somatic, and in the case of the layer 5 pyramidal cell model, also to dendritic input currents has been extensively studied in earlier studies [31, 54].

1.2.2 Network Behaviour

Noise-driven network To validate our control network model, we first performed simulations (20 subjects, 10 trials each subject, see Supplementary Section 1.1.2) with only the background noise as input and calculated the background firing rates for each population. The pyramidal cell population fired at an average rate of 7.81 Hz (standard deviation: 0.07 Hz) and the basket cell population at 6.18 Hz (standard deviation: 0.26 Hz), which is reasonably close to the range of 4.9 Hz (standard deviation: 0.5) reported for auditory cortex [20].

In order to explore the behaviour of the control network further, simulations with 20, 30 and 40 Hz drive were performed and the simulated local field potential was recorded. We explicitly went beyond purely driving the network with 40 Hz stimuli and included drive at 20 and 30 Hz, which are routinely used in experimental ASSR studies in patients with schizophrenia (e.g. [24, 55]). As can be seen in Supplementary Figure 1, the network clearly entrains to the driving frequency, the power at drive frequency is highest for 40 Hz and lowest for 20 Hz and for 20 Hz drive, a clear 40 Hz component is visible, thus, the network replicates experimental studies [24, 55] and

previous modeling studies [55, 39, 37, 47].

Furthermore, Cardin et al. [3] show that rhythmic optogenetic drive of FS cells specifically enhances power in the gamma range. In our control network model, we rhythmically drive FS cells (with a sinusoidal current instead of optogenetic drive though) with frequencies between 10 and 100 Hz, in steps of 10 Hz, while excitatory cells only receive noise drive. Similarly to Cardin et al. [3], we see that periodically stimulating FS cells enhances LFP power especially in the gamma frequency range (30-60 Hz) (Supplementary Figure 1). This not only replicates the findings of Cardin et al. [3], but also agrees with modelling findings of Vierling-Claassen et al. [54], which demonstrated that a Pyr-FS cell circuit can explain the gamma resonance seen experimentally well.

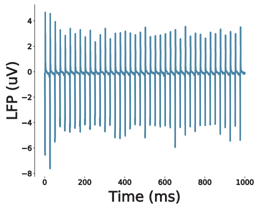

(a)

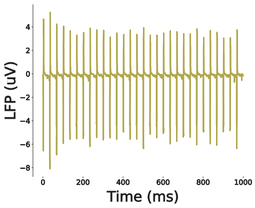

(b)

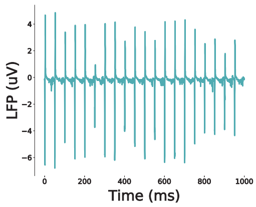

(c)

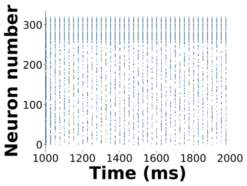

(d)

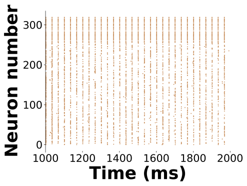

(e)

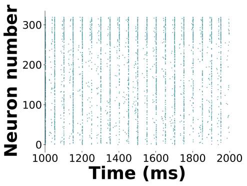

(f)

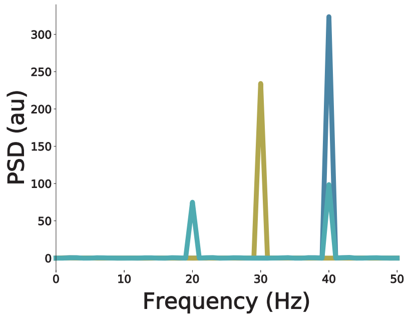

(g)

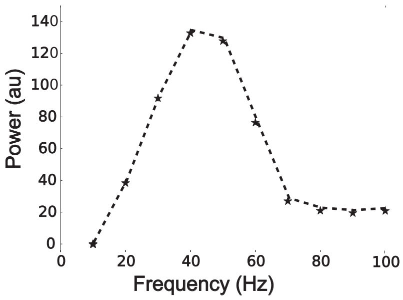

(h)

Figure 1: **Validation of the control network model.** (a) Simulated LFP in response to a 40 Hz drive. (b) Simulated LFP in response to a 30 Hz drive. (a) Simulated LFP in response to a 20 Hz drive.(d-f) Raster plots for the signals from (a-c). Neurons from 0 to 255 are excitatory pyramidal cells and from 256 to 319 are inhibitory basket cells. (g) Power spectral densities of the three signals from (a)-(c). (h) Peak power for simulations with 10-100 Hz drive to only the FS basket cell populations (Note that in the standard ASSR condition (a-d) both cell populations receive drive input).

1.2.3 Single Variant Effects

Supplementary Table 6 shows the mean differences and permutation statistics for selected single model variants and model variant combinations as well as the two synaptic alterations.

	sample size	mean difference	99% CI interval	permutation p-value
Ca7	20	-29.5	[-30.9, -28.2]	< 0.001
HCN2	20	-21.9	[-23.7, -20.1]	< 0.001
Ca74	20	-16.7	[-18.1, -15.2]	< 0.01
C1	20	-49.7	[-51.0, -48.4]	< 0.001
C2	20	-61.9	[-62.2, -60.6]	< 0.001
C3	20	-67.2	[-68.5, -65.9]	< 0.001
Reduced g_{max}	20	-88.4	[-90.0, -86.7]	< 0.001
Increased τ_{inh}	20	-181.0	[-183.0, -17.0]	< 0.001

Table 6: **Permutation test statistics**. Two-sided comparison against a shared control with a sample size of $n = 20$. 5000 bootstrap samples were taken; the confidence interval is bias-corrected and accelerated. The p values reported are the likelihoods of observing the mean differences, if the null hypothesis of zero difference is true. For each permutation p value, 5000 reshuffles of the control and test labels were performed. For more details see [18].

Several experimental [24, 55] and modeling [55, 39, 37, 40] studies have found an increase in the subharmonic 20 Hz component in response to 40 Hz drive in SCZ. Therefore, we also quantified 20 Hz power in our exploration of the single model variant effects. As can be seen in Supplementary Figure 2, single model variants had hardly any effects on the 20 Hz component.

We correlated parameter changes to gamma reduction for the model variants affecting I_{CaHVA}. As can be seen in Supplementary Figure 3 the change in evoked gamma power was positively correlated with the offset of the activation variable m but there was no significant correlation with other parameter changes.

We calculated Pearson correlation coefficients between the ratio of gamma reduction in our model and the resonance power in the delta band and the pre-pulse inhibition (PPI) thresholds from a model from previous work [34], respectively, for all 86 model variants considered here. Supplementary Figures 4 and 5 shows the scatter plots for each of the comparisons. See original manuscript for more information on the previous model.

The simulated click train experiments presented in the main manuscript implement the presentation of the individual clicks as occurring simultaneously across all neurons in the network model. This simplifying assumptions leads to very regular simulated LFP signals which make results most obvious because the peaks in the power spectrum are very clear and narrow and also the emergence of subharmonic components (as in the case of increased inhibitory synaptic time constants and a shift of power from 40 Hz to 20 Hz) can be detected more easily. However, this assumptions is certainly not completely realistic. Therefore, we also introduced jitter to the arrival times of

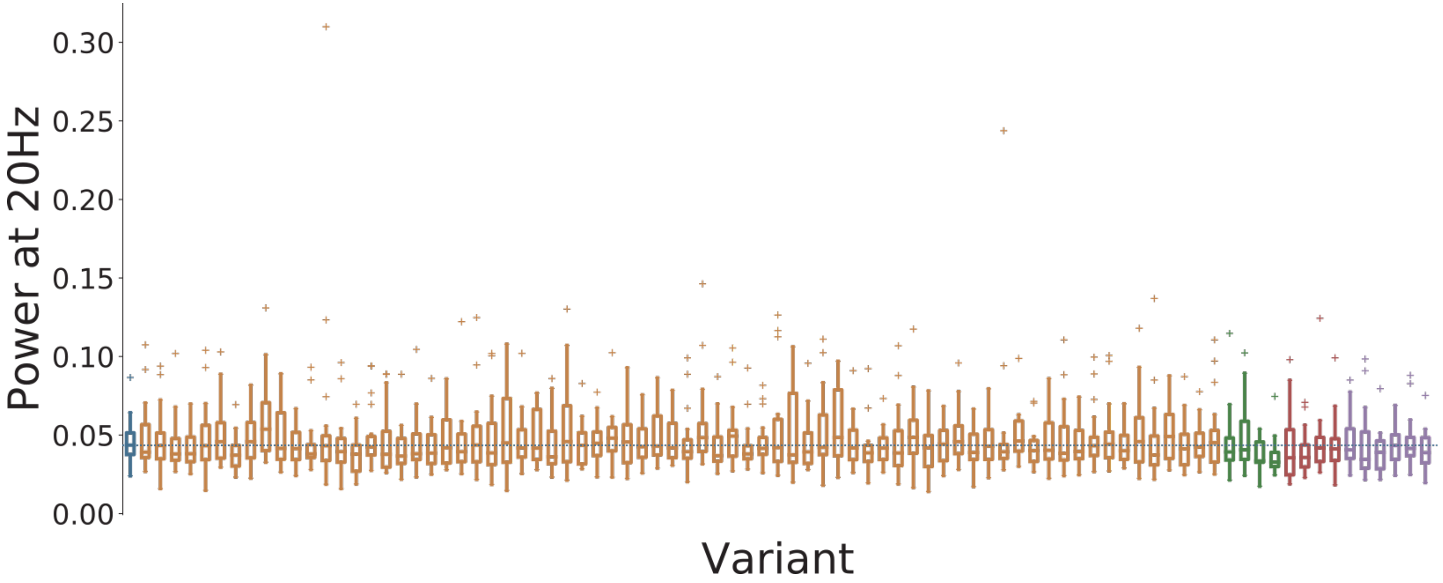

Figure 2: **Overview of the 2040 measure for all variants**. Control network in blue, Ca^{2+} channel variants affecting Ca_{HVA} in orange, affecting Ca_{LVA} in green, HCN variants in red, and SCN variants in purple. Solid lines represent the mean, box edges the 25 and 75 percentile, respectively, the whisker extend to 2 standard deviations and + depict outliers. The dashed blue line represents the control mean.

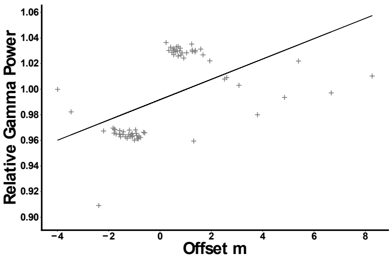

(a) **r=0.53,p<0.001**

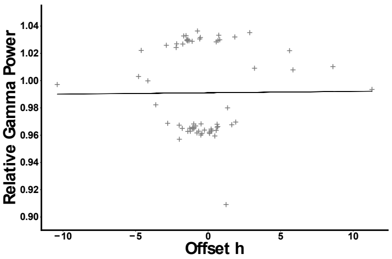

(b) r=0.01,p=0.94

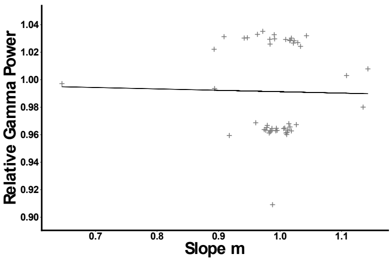

(c) r=-0.02,p=0.88

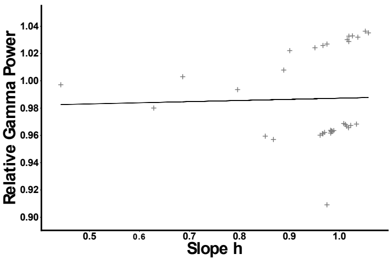

(d) r=0.03,p=0.85

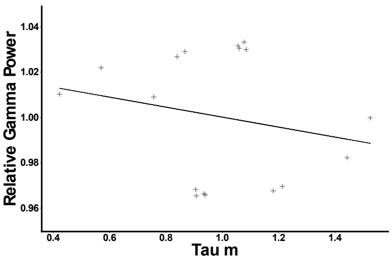

(e) r=-0.21,p=0.41

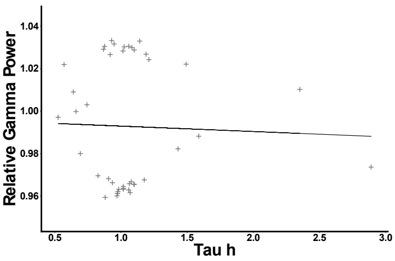

(f) r=-0.03,p=0.82

Figure 3: **Correlation of parameter changes with gamma reduction**. Pearson correlation between scaled changes of a parameter of the high-voltage activated Ca^{2+} channel and relative gamma power (i.e. the ratio of gamma power of a variant and the gamma power of the control).

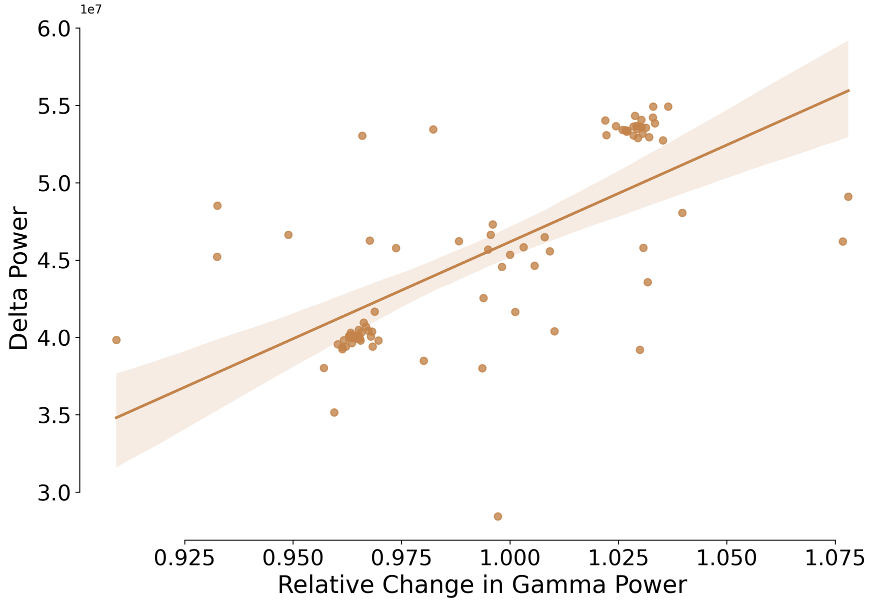

Figure 4: **Correlation of gamma reduction with resonance power in the delta band.** Pearson correlation between relative gamma power (i.e. the ratio of gamma power of a variant and the gamma power of the control) and the power in the delta of the network model from [34] in the delta resonance setup for each of the 86 variants. Scatter plot shows values for each of the 86 variants ordered as in table 3 together with a regression line of a linear model and its 95% confidence interval.

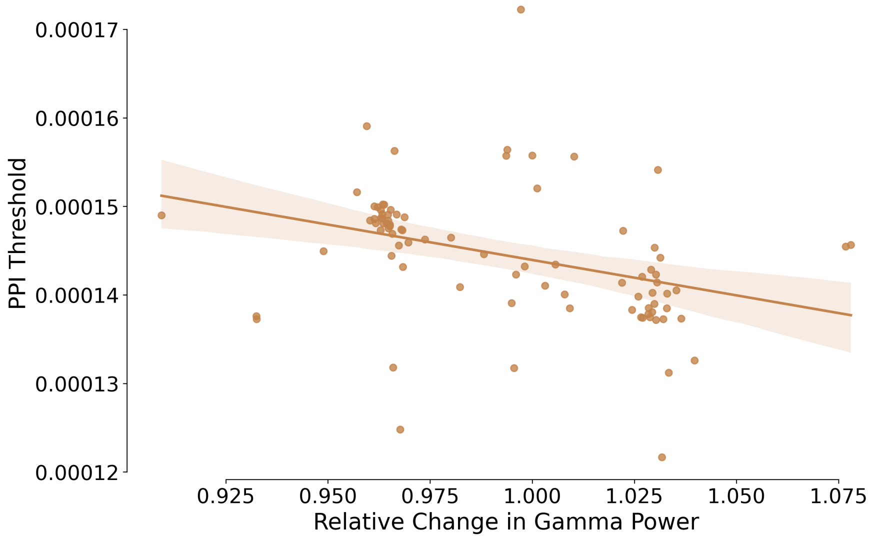

Figure 5: **Correlation of gamma reduction with PPI thresholds**. Pearson correlation between relative gamma power (i.e. the ratio of gamma power of a variant and the gamma power of the control) and the power in the PPI threshold of the network model from [34] in the PPI setup for each of the 86 variants. Scatter plot shows values for each of the 86 variants ordered as in table 3 together with a regression line of a linear model and its 95% confidence interval.

the click inputs at the individual neurons. We drew jitter from a normal distribution centered around the start of a gamma cycle (i.e. a mean of 0) with a standard deviation of 2.5 ms. In this 'jitter' condition we compared the control network to the network with the model variant 'Ca7' which demonstrated the largest reduction of gamma power in the non-jittered condition. The results are depicted in Supplementary Figure 6. The introduction of jitter results in a noisier local field potential with broader peaks ((b) and (c)) which appear more realistic than the clear and narrow peaks of the non-jittered condition. The strong reduction of power at 40 Hz for the model variant, however, is unaffected but the introduction of the jitter, demonstrating the robustness of the results.

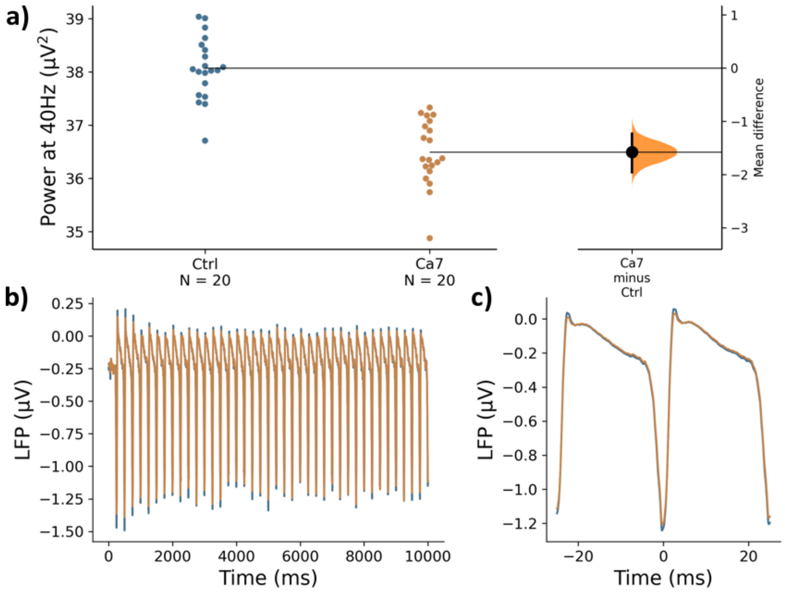

Figure 6: **Comparison of control network against model variant in the jittered input condition**. The mean difference for the comparisons of the single model variant with the strongest gamma reduction (Ca7) against the shared control are shown in the above Cumming estimation plot. The raw data is plotted on the upper axes. On the lower axes, mean differences are plotted as bootstrap sampling distributions. Each mean difference is depicted as a dot. Each 95% confidence interval is indicated by the ends of the vertical error bars. (b) The simulated LFP signal for the control network (blue) and Ca7 (yellow) is shown (c) The signal from (b) is shown, averaged over two consecutive gamma cycles. Note that in (b) and (c) the LFP signal is first averaged over all 'subjects' and 'trials'.

2 Data Availability

All code to simulate the computational model, to analyze the data and to create the figures is available at *https://github.com/ChristophMetzner/ACnet*. The model code will also be made available through ModelDB (*https://senselab.med.yale.edu/modeldb/*) upon publication. Furthermore, the model will also be included in the ASSRUnit package, which is designed for automated validation and comparison of models of ASSR deficits in psychiatric disorders [38].

References

[1] Elena AB Azizan, Hanne Poulsen, Petronel Tuluc, Junhua Zhou, Michael V Clausen, Andreas Lieb, Carmela Maniero, Sumedha Garg, Elena G Bochukova, Wanfeng Zhao, et al. Somatic mutations in atp1a1 and cacna1d underlie a common subtype of adrenal hypertension. *Nature Genetics*, 45(9):1055–1060, 2013.

[2] Gabriella Bock, Mathias Gebhart, Anja Scharinger, Wanchana Jangsangthong, Perrine Busquet, Chiara Poggiani, Simone Sartori, Matteo E Mangoni, Martina J Sinnegger-Brauns, Stefan Herzig, et al. Functional properties of a newly identified c-terminal splice variant of cav1. 3 l-type ca2+ channels. *Journal of Biological Chemistry*, 286(49):42736–42748, 2011.

[3] Jessica A Cardin, Marie Carlén, Konstantinos Meletis, Ulf Knoblich, Feng Zhang, Karl Deisseroth, Li-Huei Tsai, and Christopher I Moore. Driving fast-spiking cells

induces gamma rhythm and controls sensory responses. *Nature*, 459(7247):663–667, 2009.

[4] William A Catterall, Edward Perez-Reyes, Terrance P Snutch, and Joerg Striessnig. International union of pharmacology. xlviii. nomenclature and structure-function relationships of voltage-gated calcium channels. *Pharmacological Reviews*, 57(4):411–425, 2005.

[5] Sandrine Cestèle, Angelo Labate, Raffaella Rusconi, Patrizia Tarantino, Laura Mumoli, Silvana Franceschetti, Grazia Annesi, Massimo Mantegazza, and Antonio Gambardella. Divergent effects of the t1174s scn1a mutation associated with seizures and hemiplegic migraine. *Epilepsia*, 54(5):927–935, 2013.

[6] Sandrine Cestèle, Paolo Scalmani, Raffaella Rusconi, Benedetta Terragni, Silvana Franceschetti, and Massimo Mantegazza. Self-limited hyperexcitability: Functional effect of a familial hemiplegic migraine mutation of the Nav1.1 (SCN1A) na+ channel. *Journal of Neuroscience*, 28(29):7273–7283, 2008.

[7] Elodie Christophe, Nathalie Doerflinger, Daniel J Lavery, Zoltán Molnár, Serge Charpak, and Etienne Audinat. Two populations of layer v pyramidal cells of the mouse neocortex: development and sensitivity to anesthetics. *Journal of Neurophysiology*, 94(5):3357–3367, 2005.

[8] Jonathan M Cordeiro, Mark Marieb, Ryan Pfeiffer, Kirstine Calloe, Elena Burashnikov, and Charles Antzelevitch. Accelerated inactivation of the L-type calcium

current due to a mutation in CACNB2b underlies brugada syndrome. *Journal of Molecular and Cellular Cardiology*, 46(5):695–703, 2009.

[9] Katrin Depil, Stanislav Beyl, Anna Stary-Weinzinger, Annette Hohaus, Eugen Timin, and Steffen Hering. Timothy mutation disrupts the link between activation and inactivation in cav1. 2 protein. *Journal of Biological Chemistry*, 286(36):31557–31564, 2011.

[10] Alain Destexhe, Zachary F Mainen, and Terrence J Sejnowski. An efficient method for computing synaptic conductances based on a kinetic model of receptor binding. *Neural computation*, 6(1):14–18, 1994.

[11] Alain Destexhe, Zachary F Mainen, and Terrence J Sejnowski. Synthesis of models for excitable membranes, synaptic transmission and neuromodulation using a common kinetic formalism. *Journal of computational neuroscience*, 1(3):195–230, 1994.

[12] Salvador Dura-Bernal, Benjamin A Suter, Padraig Gleeson, Matteo Cantarelli, Adrian Quintana, Facundo Rodriguez, David J Kedziora, George L Chadderdon, Cliff C Kerr, Samuel A Neymotin, et al. Netpyne, a tool for data-driven multiscale modeling of brain circuits. *Elife*, 8:e44494, 2019.

[13] Juan Carlos Gomora, Janet Murbartián, Juan Manuel Arias, Jung-Ha Lee, and Edward Perez-Reyes. Cloning and expression of the human t-type channel cav3. 3: insights into prepulse facilitation. *Biophysical journal*, 83(1):229–241, 2002.

[14] Guillermo González-Burgos, Leonid S Krimer, Nadya V Povysheva, German Bar-

rionuevo, and David A Lewis. Functional properties of fast spiking interneurons and their synaptic connections with pyramidal cells in primate dorsolateral prefrontal cortex. *Journal of neurophysiology*, 93(2):942–953, 2005.

[15] Guillermo Gonzalez-Burgos and David A Lewis. Gaba neurons and the mechanisms of network oscillations: implications for understanding cortical dysfunction in schizophrenia. *Schizophrenia bulletin*, 34(5):944–961, 2008.

[16] Etay Hay, Sean Hill, Felix Schürmann, Henry Markram, and Idan Segev. Models of neocortical layer 5b pyramidal cells capturing a wide range of dendritic and perisomatic active properties. *PLoS Computational Biology*, 7:e1002107, 2011.

[17] Michael L Hines and Nicholas T Carnevale. The neuron simulation environment. *Neural computation*, 9(6):1179–1209, 1997.

[18] Joses Ho, Tayfun Tumkaya, Sameer Aryal, Hyungwon Choi, and Adam Claridge-Chang. Moving beyond p values: Everyday data analysis with estimation plots. *Nature Methods*, 16:565–566, 2019.

[19] Annette Hohaus, Stanislav Beyl, Michaela Kudrnac, Stanislav Berjukow, Eugen N Timin, Rainer Marksteiner, Marion A Maw, and Steffen Hering. Structural determinants of l-type channel activation in segment iis6 revealed by a retinal disorder. *Journal of Biological Chemistry*, 280(46):38471–38477, 2005.

[20] Tomáš Hromádka, Michael R DeWeese, and Anthony M Zador. Sparse representation of sounds in the unanesthetized auditory cortex. *PLoS biology*, 6(1), 2008.

[21] Dan Hu, HECTOR BARAJAS-MARTINEZ, Vladislav V Nesterenko, Ryan Pfeiffer, Alejandra Guerchicoff, Jonathan M Cordeiro, Anne B Curtis, Guido D Pollevick, Yuesheng Wu, Elena Burashnikov, et al. Dual variation in scn5a and cacnb2b underlies the development of cardiac conduction disease without brugada syndrome. *Pacing and clinical electrophysiology*, 33(3):274–285, 2010.

[22] Takahiro M Ishii, Noriyuki Nakashima, and Harunori Ohmori. Tryptophan-scanning mutagenesis in the s1 domain of mammalian hcn channel reveals residues critical for voltage-gated activation. *Journal of Physiology*, 579(2):291–301, 2007.

[23] Michaela Kudrnac, Stanislav Beyl, Annette Hohaus, Anna Stary, Thomas Peterbauer, Eugen Timin, and Steffen Hering. Coupled and independent contributions of residues in IS6 and IIS6 to activation gating of CaV1.2. *Journal of Biological Chemistry*, 284(18):12276–12284, 2009.

[24] Jun Soo Kwon, Brian F O'Donnell, Gene V Wallenstein, Robert W Greene, Yoshio Hirayasu, Paul G Nestor, Michael E Hasselmo, Geoffrey F Potts, Martha E Shenton, and Robert W McCarley. Gamma frequency–range abnormalities to auditory stimulation in schizophrenia. *Archives of general psychiatry*, 56(11):1001–1005, 1999.

[25] S Hong Lee, Teresa R DeCandia, Stephan Ripke, Jian Yang, Patrick F Sullivan, Michael E Goddard, Matthew C Keller, Peter M Visscher, Naomi R Wray, Schizophrenia Psychiatric Genome-Wide Association Study Consortium, et al. Estimating the proportion of variation in susceptibility to schizophrenia captured by common snps. *Nature Genetics*, 44(3):247–250, 2012.

[26] Heinte Lesso and Ronald A Li. Helical secondary structure of the external s3-s4 linker of pacemaker (hcn) channels revealed by site-dependent perturbations of activation phenotype. *Journal of Biological Chemistry*, 278(25):22290–22297, 2003.

[27] Andreas Lieb, Anja Scharinger, Simone Sartori, Martina J Sinnegger-Brauns, and Jörg Striessnig. Structural determinants of cav1. 3 l-type calcium channel gating. *Channels*, 6(3):197–205, 2012.

[28] Sabine Link, Marcel Meissner, Brigitte Held, Andreas Beck, Petra Weissgerber, Marc Freichel, and Veit Flockerzi. Diversity and developmental expression of L-type calcium channel $\beta 2$ proteins and their influence on calcium current in murine heart. *Journal of Biological Chemistry*, 284(44):30129–30137, 2009.

[29] Jenny Y Ma, William A Catterall, and Todd Scheuer. Persistent sodium currents through brain sodium channels induced by g protein β γ subunits. *Neuron*, 19(2):443–452, 1997.

[30] Zachary F Mainen and Terrence J Sejnowski. Influence of dendritic structure on firing pattern in model neocortical neurons. *Nature*, 382(6589):363–366, 1996.

[31] T Mäki-Marttunen, G Halnes, A Devor, C Metzner, A M Dale, O A Andreassen, and G T Einevoll. A stepwise neuron model fitting procedure designed for recordings with high spatial resolution: Application to layer 5 pyramidal cells. *Journal of Neuroscience Methods*, 273:264–283, 2018.

[32] Tuomo Mäki-Marttunen, Geir Halnes, Anna Devor, Christoph Metzner, Anders M

Dale, Ole A Andreassen, and Gaute T Einevoll. A stepwise neuron model fitting procedure designed for recordings with high spatial resolution: application to layer 5 pyramidal cells. *Journal of neuroscience methods*, 293:264–283, 2018.

[33] Tuomo Mäki-Marttunen, Geir Halnes, Anna Devor, Aree Witoelar, Francesco Bettella, Srdjan Djurovic, Yunpeng Wang, Gaute T. Einevoll, Ole A. Andreassen, and Anders M. Dale. Functional effects of schizophrenia-linked genetic variants on intrinsic single-neuron excitability: A modeling study. *Biological Psychiatry: Cognitive Neuroscience and Neuroimaging*, 1:49–59, 2016.

[34] Tuomo Mäki-Marttunen, Florian Krull, Francesco Bettella, Espen Hagen, Solveig Næss, Torbjørn V Ness, Torgeir Moberget, Torbjørn Elvsåshagen, Christoph Metzner, Anna Devor, et al. Alterations in schizophrenia-associated genes can lead to increased power in delta oscillations. *Cerebral Cortex*, 29(2):875–891, 2019.

[35] Massimo Mantegazza, Antonio Gambardella, Raffaella Rusconi, Emanuele Schiavon, Ferdinanda Annesi, Rita Restano Cassulini, Angelo Labate, Sara Carrideo, Rosanna Chifari, Maria Paola Canevini, et al. Identification of an nav1.1 sodium channel (scn1a) loss-of-function mutation associated with familial simple febrile seizures. *Proceedings of the National Academy of Sciences of the United States of America*, 102(50):18177–18182, 2005.

[36] Enrique Massa, Kevin M Kelly, David I Yule, Robert L MacDonald, and Michael D Uhler. Comparison of fura-2 imaging and electrophysiological analysis of murine calcium channel alpha 1 subunits coexpressed with novel beta 2 subunit isoforms. *Molecular Pharmacology*, 47(4):707–716, 1995.

[37] Christoph Metzner. [Re] Modeling GABA Alterations in Schizophrenia: A Link Between Impaired Inhibition and Gamma and Beta Auditory Entrainment. *Re-Science*, 3(1):6, August 2017.

[38] Christoph Metzner, Tuomo Mäki-Marttunen, Bartosz Zurowski, and Volker Steuber. Modules for automated validation and comparison of models of neurophysiological and neurocognitive biomarkers of psychiatric disorders: Assrunit—a case study. *Computational Psychiatry*, 2:74–91, 2018.

[39] Christoph Metzner, Achim Schweikard, and Bartosz Zurowski. Multifactorial modeling of impairment of evoked gamma range oscillations in schizophrenia. *Frontiers in computational neuroscience*, 10:89, 2016.

[40] Christoph Metzner, Bartosz Zurowski, and Volker Steuber. the role of parvalbumin-positive interneurons in auditory steady-state response deficits in schizophrenia. *Scientific Reports*, 9(1):1–16, 2019.

[41] A Munoz, Timothy M Woods, and Edward G Jones. Laminar and cellular distribution of ampa, kainate, and nmda receptor subunits in monkey sensory–motor cortex. *Journal of Comparative Neurology*, 407(4):472–490, 1999.

[42] Janet Murbartián, Juan Manuel Arias, and Edward Perez-Reyes. Functional impact of alternative splicing of human t-type cav3. 3 calcium channels. *Journal of neurophysiology*, 92(6):3399–3407, 2004.

[43] Chaelon IO Myme, Ken Sugino, Gina G Turrigiano, and Sacha B Nelson. The nmda-to-ampa ratio at synapses onto layer 2/3 pyramidal neurons is conserved

across prefrontal and visual cortices. *Journal of neurophysiology*, 90(2):771–779, 2003.

[44] Alberto Pérez-Alvarez, Alicia Hernández-Vivanco, Jose Carlos Caba-González, and Almudena Albillos. Different roles attributed to cav1 channel subtypes in spontaneous action potential firing and fine tuning of exocytosis in mouse chromaffin cells. *Journal of Neurochemistry*, 116(1):105–121, 2011.

[45] Alexandra Pinggera, Andreas Lieb, Bruno Benedetti, Michaela Lampert, Stefania Monteleone, Klaus R Liedl, Petronel Tuluc, and Jörg Striessnig. Cacna1d de novo mutations in autism spectrum disorders activate cav1. 3 l-type calcium channels. *Biological psychiatry*, 77(9):816–822, 2015.

[46] Barbara Rosati and David McKinnon. Regulation of ion channel expression. *Circulation Research*, 94(7):874–883, 2004.

[47] Peter J Siekmeier et al. Development of antipsychotic medications with novel mechanisms of action based on computational modeling of hippocampal neuropathology. *PloS one*, 8(3), 2013.

[48] Nathan G Skene, Julien Bryois, Trygve E Bakken, Gerome Breen, James J Crowley, Héléna A Gaspar, Paola Giusti-Rodriguez, Rebecca D Hodge, Jeremy A Miller, Ana B Muñoz-Manchado, et al. Genetic identification of brain cell types underlying schizophrenia. *Nature genetics*, 50(6):825–833, 2018.

[49] Kevin M Spencer. The functional consequences of cortical circuit abnormalities

on gamma oscillations in schizophrenia: insights from computational modeling. *Frontiers in human neuroscience*, 3:33, 2009.

[50] Anna Stary, Michaela Kudrnac, Stanislav Beyl, Annette Hohaus, Eugen Timin, Peter Wolschann, H Robert Guy, and Steffen Hering. Molecular dynamics and mutational analysis of a channelopathy mutation in the iis6 helix of cav1. 2. *Channels*, 2(3):216–223, 2008.

[51] Bao Zhen Tan, Fengli Jiang, Ming Yeong Tan, Dejie Yu, Hua Huang, Yiru Shen, and Tuck Wah Soong. Functional characterization of alternative splicing in the c terminus of l-type cav1. 3 channels. *Journal of Biological Chemistry*, 286(49):42725–42735, 2011.

[52] Zhen Zhi Tang, Mui Cheng Liang, Songqing Lu, Dejie Yu, Chye Yun Yu, David T Yue, and Tuck Wah Soong. Transcript scanning reveals novel and extensive splice variations in human l-type voltage-gated calcium channel, cav1. 2 $\alpha 1$ subunit. *Journal of Biological Chemistry*, 279(43):44335–44343, 2004.

[53] Kaate RJ Vanmolkot, Elena Babini, Boukje de Vries, Anine H Stam, Tobias Freilinger, Gisela M Terwindt, Lisa Norris, Joost Haan, Rune R Frants, Nabih M Ramadan, et al. The novel p.L1649Q mutation in the SCN1A epilepsy gene is associated with familial hemiplegic migraine: genetic and functional studies. *Human Mutation*, 28(5):522–522, 2007.

[54] Dorea Vierling-Claassen, Jessica Cardin, Christopher I Moore, and Stephanie R Jones. Computational modeling of distinct neocortical oscillations driven by cell-

type selective optogenetic drive: separable resonant circuits controlled by low-threshold spiking and fast-spiking interneurons. *Frontiers in human neuroscience*, 4:198, 2010.

[55] Dorea Vierling-Claassen, Peter Siekmeier, Steven Stufflebeam, and Nancy Kopell. Modeling gaba alterations in schizophrenia: a link between impaired inhibition and altered gamma and beta range auditory entrainment. *Journal of neurophysiology*, 99(5):2656–2671, 2008.

[56] Linda Volkers, Kristopher M Kahlig, Nienke E Verbeek, Joost HG Das, Marjan JA van Kempen, Hans Stroink, Paul Augustijn, Onno van Nieuwenhuizen, Dick Lindhout, Alfred L George, et al. Nav1. 1 dysfunction in genetic epilepsy with febrile seizures-plus or dravet syndrome. *European Journal of Neuroscience*, 34(8):1268–1275, 2011.

[57] Konstantin Wemhöner, Tatyana Kanyshkova, Nicole Silbernagel, Juncal Fernandez-Orth, Stefan Bittner, Aytug K Kiper, Susanne Rinné, Michael F Netter, Sven G Meuth, Thomas Budde, et al. An n-terminal deletion variant of hcn1 in the epileptic wag/rij strain modulates hcn current densities. *Frontiers in molecular neuroscience*, 8:83, 2015.

[58] William RJ Whitaker, Jeffrey J Clare, Andrew J Powell, Yu Hua Chen, Richard LM Faull, and Piers C Emson. Distribution of voltage-gated sodium channel α-subunit and β-subunit mrnas in human hippocampal formation, cortex, and cerebellum. *Journal of Comparative Neurology*, 422(1):123–139, 2000.

[59] Q. Zhang, V. Timofeyev, H. Qiu, L. Lu, N. Li, A. Singapuri, C. L. Torado, H. S. Shin, and N. Chiamvimonvat. Expression and roles of Cav1.3 (α1D) L-type Ca2+ channel in atrioventricular node automaticity. *Journal of Molecular and Cellular Cardiology*, 50(1):194–202, 2011.
